# Glial Cells as Key Regulators in Neuroinflammatory Mechanisms Associated with Multiple Sclerosis

**DOI:** 10.3390/ijms25179588

**Published:** 2024-09-04

**Authors:** Styliani Theophanous, Irene Sargiannidou, Kleopas A. Kleopa

**Affiliations:** 1 Neuroscience Department, The Cyprus Institute of Neurology and Genetics, 2371 Nicosia, Cyprus; stylianith@cing.ac.cy (S.T.); irenes@cing.ac.cy (I.S.); 2 Center for Multiple Sclerosis and Related Disorders, The Cyprus Institute of Neurology and Genetics, 2371 Nicosia, Cyprus

**Keywords:** astrocytes, blood–brain barrier, communication, demyelination, inflammation, microglia, neurodegeneration, oligodendrocytes, remyelination

## Abstract

Even though several highly effective treatments have been developed for multiple sclerosis (MS), the underlying pathological mechanisms and drivers of the disease have not been fully elucidated. In recent years, there has been a growing interest in studying neuroinflammation in the context of glial cell involvement as there is increasing evidence of their central role in disease progression. Although glial cell communication and proper function underlies brain homeostasis and maintenance, their multiple effects in an MS brain remain complex and controversial. In this review, we aim to provide an overview of the contribution of glial cells, oligodendrocytes, astrocytes, and microglia in the pathology of MS during both the activation and orchestration of inflammatory mechanisms, as well as of their synergistic effects during the repair and restoration of function. Additionally, we discuss how the understanding of glial cell involvement in MS may provide new therapeutic targets either to limit disease progression or to facilitate repair.

## 1. Introduction

Multiple sclerosis (MS) is the most common demyelinating neurodegenerative disease that affects young adults, and it is characterized by neuroinflammation of the central nervous system (CNS), during which the infiltrating peripheral immune cells cause damage to the myelin and axons of the neuronal cells [1]. MS symptoms and clinical signs are variable depending on which areas of the brain and spinal cord are affected, and the disease course is heterogeneous as it may follow different degrees of severity and progression. MS is broadly categorized into the following three phenotypes: relapsing-remitting MS (RRMS), primary progressing MS (PPMS), and secondary progressive MS (SPMS), depending on the disease course. The pathological hallmark of the disease, plaque formation, is evident in multiple CNS areas, including the cerebral white matter (WM) and gray matter (GM), brainstem, spinal cord, and optic nerve. The evaluation of the lesions with MRI has proven to be a sensitive method to diagnose a patient. Gadolinium enhancement on the MRI is a marker of active and focal inflammation as the dye penetrates the broken blood–brain barrier (BBB) [2].

The loss of BBB permeability is due to the secretion of highly harmful reactive oxygen and nitrogen species (ROS, NOS) [3] from the perivascular astrocytes resulting in edema formation and infiltration of immune cells into the CNS [4]. As the extracellular matrix (ECM) relaxes, there is a degradation of the molecules including elastin, generating elastin-derived peptides like serine proteases, cysteine proteases, and matrix metalloproteinases (MMPs) [5]. Elastin-derived peptides have been shown to interact with various receptors, leading to the activation of intracellular signaling pathways like inflammation and cell proliferation, especially with glial cells, and have also been implicated in various neurodegenerative diseases [6]. Finally, ECM proteins released were shown to be deposited in lesional and perilesional areas and, since they are known potent pro-inflammatory molecules, they are thought to contribute to disease severity and remyelination failure [7].

Aside from the disruption of the BBB, T cells and, in particular, the CD4+ T cells play a key role in the disease pathology. When naïve CD4+ T cells are exposed to interleukin (IL)-12, they are polarized to the T helper (Th) 1 phenotype and produce cytokines including interferon gamma (IFN-γ) and tumor necrosis factor alpha (TNFα). In the presence of transforming growth factor (TGF)-β and IL6, naïve CD4+ T cells become Th17 cells and secrete IL-17 and granulocyte macrophage colony stimulating factor (GM-CSF) that are pro-inflammatory in many animal models. High levels of circulating IL-17 were measured in the active MS patients compared to the healthy controls, suggesting its involvement in CNS inflammation [8]. In addition to the T cells, B cells are also essential players in MS pathogenesis. B cells not only produce cytokines that are proinflammatory (IFN-γ) and regulatory (IL-10), but they also present antigens to T cells [9]. Moreover, B cells form ectopic structures, follicles, in MS patients that express CD20 [10]. Treatments with anti-CD20 monoclonal antibodies have been proven to be very effective and lead to disease stabilization.

**Figure 1:**
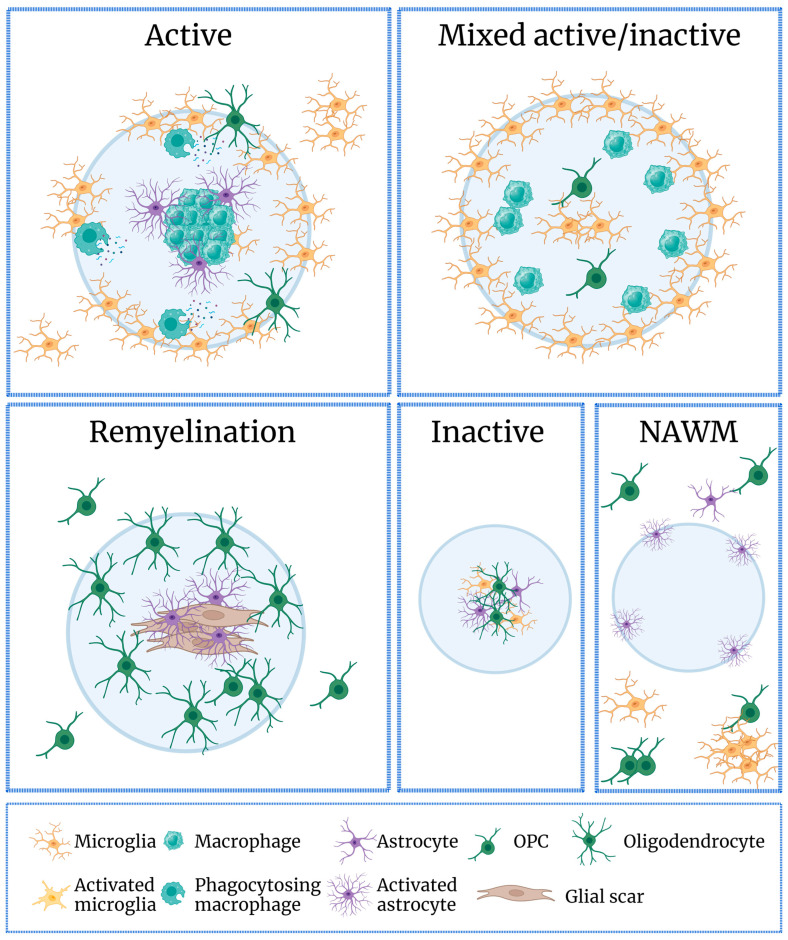
Glial cells populate lesions in a heterogeneous manner during the course of MS lesions. During the course of the disease, lesions change in respect to glial cell population. Active lesions contain macrophages at the center, while astrocytosis starts to be present. The rim of the lesion is populated with microglia which recruit other inflammatory cells to the site, as well as myelin-phagocytosing macrophages. Mixed active/inactive lesions do not show significant astrocyte population, while there is distinct formation of microglia around the rim. Additionally, there is recruitment of OPCs. Remyelination can be seen as a shadow plaque, where oligodendrocytes slowly initiate the repair process. Astrocytes and glial scar formation are clearly present at the center of the lesion. Inactive lesions show some degree of scar with few astrocytes and microglia present. Last but not least, recruitment of OPCs, and microglia nodules, can be seen in the NAWM, even in significant distance from the lesion site. OPC, Oligodendrocyte precursor cells; NAWM, normal-appearing white matter (figure was created with BioRender.com).

During early MS, glial activation plays a key role in the initial reactive process, and is thought to be key in determining the fate of the disease progression [11]. MS lesions have different characteristics depending on their state of activation and position, as well as regional glial subtype diversity [12] (Figure 1). Active lesions are characterized by reduced oligodendrocytes, the accumulation of microglia and macrophages throughout the lesion area, and myeloid cells (granulocytes and monocytes) phagocytosing myelin proteins [13], for which recent evidence has suggested that the vast majority is resident-derived microglia [14]. Additionally, hypertrophic astrocytes, which express proinflammatory cytokines, chemokines, and remyelination signaling molecules, are present and act as phagocytes in MS lesions [15,16]. Astroglial scar formation is limited or absent at this stage [17]. Chronic active lesions, where complete demyelination is evident, have a hypocellular lesion core, a sharp edge, with microglia and macrophages to be limited to the lesion border with increased major histocompatibility complex (MHC) presentation [14]. Chronic active lesions also contain myeloid cells which secrete proinflammatory molecules [18]. An astroglial scar is present at the core, while hypertrophic astrocytes surround the lesion border. Most importantly, remyelination can be seen at the border, indicating the healing process [19]. In contrast, in chronic inactive lesions, microglia and macrophages are almost absent [20], as is remyelination. In remyelinated shadow plaques, there is limited axonal degeneration, some astroglial scarring, few microglia and macrophages, and reappearance of oligodendrocytes [21].

At present, MS management strategies mainly include disease-modifying therapies (DMTs) and monoclonal antibodies. Therapies focus on treating acute attacks, improving symptoms and reducing inflammatory activity to prevent further relapses using immunomodulatory and/or immunosuppressive molecules, while little progress has been made into treating relapses themselves. Additionally, in cases of flares-ups, corticosteroids such as methylprednisolone, which has anti-inflammatory and immunosuppressive effects, may be prescribed to limit the inflammatory response, as well as symptomatic medications for other disease manifestations. Nevertheless, depending on the disease course and clinical picture, different personalized therapeutic strategies are followed. While therapeutic strategies show sufficient efficacy in controlling or suppressing disease activity, they mainly concentrate on the initial dominating inflammation driven by adaptive immunity and not on the chronic inflammation driven by CNS innate immunity and the associated neurodegeneration. Efficient remyelinating therapy, especially at early stages of MS, may halt long-term axonal degeneration and possibly prevent secondary progression [22]. This underscores the importance of investigating the glial cell involvement in the inflammatory processes and repair.

MS has been extensively studied using experimental animal models to both investigate pathological mechanisms as well as assess treatment efficiency. The most studied animal model is the immune-mediated experimental autoimmune encephalomyelitis (EAE) mouse model as it replicates the inflammation, demyelination, gliosis, and axonal loss that are present in MS [23]. While it provides the opportunity to understand the consequences of the inflammatory response in the CNS, it shows significant differences compared to the human disease and does not always cause myelin loss [24]. The viral-induced demyelination mouse model caused by the Theiler’s murine encephalomyelitis virus (TMEV) is also used, as the virus-induced pathology is very similar to the human chronic progressive MS; however, the persistent viral infection of the CNS is not commonly seen in the human disease. Since the neuroinflammatory models of MS, including EAE and TMEV, have limited application in the study of the remyelination process and myelin repair, over the last decades, toxin-induced models such as cuprizone and lysolecithin (LPC) are increasingly used to study demyelination and remyelination in the CNS. Overall, the different animal models of MS, despite their limitations in not recapitulating all the pathological and clinical manifestations of the disease, they provide insights into the mechanisms of disease initiation and progression and offer useful tools for testing the safety and efficacy of novel therapeutics.

The proper immune system function is an absolute prerequisite in the regeneration process via tissue damage healing and the initiation of the repair process through cell-autonomous and non-cell-autonomous machinery. Many factors control this sequence of events, including reactive astrocytes and microglia, the demyelinated axons themselves, as well as various inflammatory molecules. The degree of remyelination varies in MS lesions, while the failure of remyelination is a common finding in MS lesions and it seems to be linked to inadequate activation, recruitment, and/or differentiation of oligodendrocyte precursor cells (OPCs) [25] and the presence of significant inflammation [26,27].

The biological pathways and therapeutic potential of remyelination efficiency have gained increasing interest over the past decades. Enhancement of endogenous remyelination could be one of the most promising interventions to delay, prevent, or reverse disease progression, and several candidate remyelination targets have emerged, including macrophage-induced inflammation antioxidants and nanoparticles [28]. As remyelination is a complex multifactorial process, a fine-tuning of the coordination of immune cell responses is needed to acquire a regulated reaction in terms of magnitude and duration of the response [29]. Glial cells are key regulators in the orchestration of such activity in the CNS, and dissecting the functional mechanisms behind their interactions is key to both understand and target these responses.

This review aims to address the underlying pathological mechanisms by which glial cells contribute to the inflammatory process during the course of MS and how these cells have increasing potential to be targeted for future therapeutic interventions. More and more evidence has implicated oligodendrocytes, the key cells involved in myelination, in immune reactivity modulation; while their exact role has not yet been thoroughly investigated, they seem to have an active role in orchestrating the inflammatory process. Additionally, the role of astrocytes and microglia in their contribution to MS will be discussed, as they have a known role in immune mechanisms. Additionally, they show distinct patterns in and around lesions, possibly predicting disease progression. Finally, glial cell function targeting will be addressed as potential modulators of the disease course, especially during progressive MS.

### Glial Cell Function and Communication

The CNS is comprised of neurons and glia, which include oligodendrocytes, microglia, astrocytes, and ependymal cells. Initially, they were regarded as passive cells, serving the physical support of neurons. However, their crucial role in the support and protection of neurons and their axons was subsequently identified, as well as their regulatory role in the efficient communication between all cells of the CNS, the maintenance of homeostasis, the facilitation of synapse formation and neurotransmission, and the detection of inflammation and tissue damage [30]. Glial cells are highly plastic and, by altering their phenotype, they can adjust to their environmental needs. During early MS, it is considered that glial cell activation is mainly triggered by autoimmune responses; however, the persistence of their activation, independent of autoimmune interactions, is key to the progression of the disease.

Intercellular communication is crucial for the normal function of the nervous system. Glia and neurons communicate via the bidirectional exchange of ions, neurotransmitters, metabolites, cell adhesion molecules, and extracellular vesicles [31]. Moreover, oligodendrocyte communication with the axon is crucial for the preservation of the axon survival and proper myelination, while oligodendrocytes promote neuronal survival via secretion of neurotrophic factors including insulin-like growth factor 1 (IGF-1) and glial cell line-derived neurotrophic factor (GDNF) [32]. However, disturbed axo-glial communication has been recognized in MS normal-appearing white matter (NAWM) and correlates to microglial inflammation [33].

The bi-directional exchange between glial cells regulates and controls their function, migration, and activity, and therefore treatments to block or induce their activation may prove effective for various immune-mediated inflammatory diseases, including MS. Glial communication is a complex, multifactorial process. Oligodendrocytes, astrocytes, and microglia have interdependent functions, exerting both beneficial and detrimental effects in MS degeneration. They facilitate various processes by secreting signaling molecules like cytokines and chemokines and therefore play a key role in the regulation of acute and chronic inflammation.

Glia cells communicate with other glia cells via the intercellular diffusion of chemical messengers [34] to maintain homeostasis and regulate synaptic transmission during development and various pathologies. Glial–glial interactions are recognized to be vital regulators in myelin health, especially astrocyte–oligodendrocyte crosstalk during myelination. The survival of mature oligodendrocytes is also dependent on astrocyte function by a downregulation of the nuclear factor erythroid 2-related factor 2 (Nrf2) pathway and an upregulation of cholesterol synthesis/export [35]. Moreover, astrocytes induce OPC proliferation, which in turn upregulates the factors involved in the tightening of the BBB as well as the regulatory function of astrocytes. Oligodendrocyte–astrocyte crosstalk exploration may provide novel insights in controlling their modulatory effects and potential therapeutic targets.

Crosstalk between astrocytes and microglia is an emerging topic of interest. It is maintained via the secretion of mediators such as growth factors, cytokines and chemokines, NOS, ROS and metabolic mediators, and others [36], and has a potent role as disease modulators [37]. However, they are modulated by inflammatory insults as microglia regulate the innate functions of astrocytes, their pro- or anti-inflammatory phenotype, and vice versa [38]. Additionally, astrocytes modulate microglial migration and phagocytic activity. Moreover, astrocyte–microglia crosstalk may be induced through the gut–brain axis after the metabolites of dietary tryptophan act directly on microglia, and the production of vascular endothelial growth factor (VEGF)-β and tumor growth factor (TGF)-α regulate astrocyte activation [39]. Astrocyte–microglia communication is also essential during injury to support neuronal survival.

A correlation between the microglial activity and oligodendrocyte damage has been established, as a wide range of inflammatory molecules have been detected in MS lesions [40]. NO production is induced by the presence of IFN-γ, TNF-α, and IL-1β secreted by microglia under stress. LPC-induced microglial activation leads to the secretion of molecules that mediate the arrest of OPC proliferation and the induction of OPC death, as well as the secretion of extracellular vesicles in MS lesions [41].

Glial cell function depends on intra- and intercellular communication mediated by gap junction (GJ) channels that connect the cytoplasm of adjacent cells and are permeable to ions and various small molecules including second messengers and metabolites [42]. GJs expressed by glial cells, and mainly astrocytes, create an intricate network named the ‘panglial syncytium’. Oligodendrocytes express Connexin (Cx)-47, Cx32, and Cx29 [43], while astrocytes express Cx43, Cx30, and Cx26 [44]. Cx47 is normally expressed in all oligodendrocytes in the CNS [45,46]; however, Cx32 is expressed only in the oligodendrocytes myelinating large diameter axons [43]. Oligodendrocyte–astrocyte connectivity, crucial for myelination and demyelination process, is formed mainly by the Cx47/Cx43 channels and, to a lesser extent, by Cx32/Cx30, and the level of coupling is highly sensitive to different signaling pathways [47].

Myelin integrity and function depends on the proper function and communication of the glial cells and connecting neurons [48]. As previously mentioned, glial cells communicate by receiving signals from neighboring cells via the Cx-formed GJ channels, which modulate a coordinated response. Previous studies have shown alterations in glial GJ expression in the brain of postmortem MS patients, both in the NAWM [49] and GM [50], while similar results were reproduced in the EAE mouse model of MS [51]. Oligodendrocyte GJ connectivity was shown to have a regulatory role in inflammatory demyelination, since the ablation of Cx47 or Cx32 led to increased EAE severity compared to the wildtype (WT) [52], while EAE induction in Cx47^−/−^ mice led to the dysregulation of the blood–spinal cord barrier (BSCB) [53]. Additionally, the ablation of Cx43 promoted remyelination by containing local inflammation [54], while the inducible conditional knockout of Cx47 increased inflammatory activation upon the induction of EAE [55]. Finally, molecules that serve as GJ blockers have been investigated for the treatment of neurodegenerative diseases [56,57,58].

## 2. Oligodendrocytes and Neuroinflammation

Oligodendrocytes are the cells responsible for forming myelin and providing insulation to the axons by ensheathing them with multiple concentric layers of membrane. The assembly of a tightly packaged membrane to form a compact myelin layering allows for the electrical insulation of the axons, increased velocity of propagation, as well as reduced axonal energy consumption [59]. Myelin compartments, called internodes, are formed with periodic interruptions, creating the nodes of Ranvier, which, by being highly enriched in ion channels, mainly clusters of voltage-gated sodium (Na^+^) channels, facilitate the propagation of the action potential via saltatory conduction. In unmyelinated axons, the action potential travels continuously, making the propagation slower, further indicating the crucial role of myelin. Myelin is composed of water, lipids, and protein molecules, including myelin basic protein (MBP), which is expressed on the cytoplasmic surface of the plasma membrane, the surface marker myelin oligodendrocyte protein (MOG), myelin-associated glycoprotein (MAG), and the transmembrane proteolipid protein (PLP) [60]. These are the main targets of autoimmune attack in MS [61]. Myelin not only facilitates saltatory conduction, but it is also important to support the axon by regulating the axonal cytoskeleton and transport of molecules and to protect it from damage [62]. Damage to myelin leads to demyelination and eventually axonal degeneration, which is the main feature of MS clinical manifestations [63].

Oligodendrocytes derive from OPCs, which, during development, differentiate and mature into myelinating oligodendrocytes via a tightly controlled process of activation and repression of transcription and growth factors, and then spread evenly into the GM and WM of the CNS. However, the adult CNS maintains a number of undifferentiated OPCs responsible for the production of myelinating oligodendrocytes during adulthood [61], with the white matter having a slightly higher abundance of OPCs than the grey matter [64]. When axonal myelination is needed, OPCs migrate from their site of origin to the developing WM tracts in a crawling mode along the blood vessels, a process highly dependent on the Wnt signaling [65,66], leading to an excessive pool of progenitors. Oligodendrocyte generation dynamics remains a crucial step into understanding the myelination process [67].

Recent technological advances have allowed for a deeper and more delicate classification of oligodendrocytes, in relation to their differentiation state, developmental origin, and expression site. Single-nucleus and single-cell RNA sequencing (snRNA-seq, scRNA-seq) from the WM of a postmortem human brain has shown that specific sub-clusters of oligodendroglia are under-represented in MS tissue compared to the healthy controls, while others are over-represented [68,69]. As the maturation state determines the mechanism of cell death that oligodendrocytes undergo in response to insult [70], these differences indicate that different functional states of oligodendrocytes are present upon tissue injury. A better understanding of their role can lead to new ways of treatment regimens [71].

Oligodendrocytes are thought to be the most vulnerable cells of the CNS due to their highly complex architecture, unique differentiation program of proliferation, migration, and myelination, as well as high metabolic demands [72,73]. They are affected by various stimuli from either neighboring astrocytes and microglia or the immune cells, which may trigger oligodendrocyte injury or apoptosis. Oligodendrocytes express different cytokines, chemokines, antigen-presenting molecules (APCs), and complement receptors and have an active role in the immune modulation in the CNS. Additionally, they assess their microenvironment by extending filopodia, especially in response to inflammatory cues [74], and migrate short distances [75].

### 2.1. Oligodendrocytic Immunomodulatory Role

**Figure 2:**
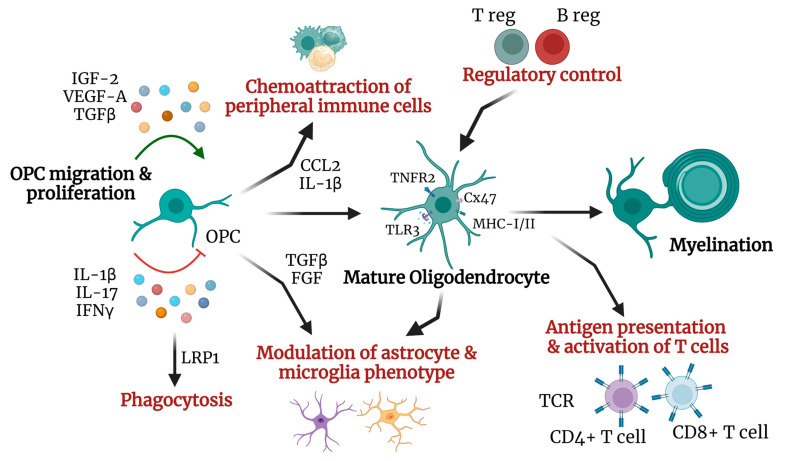
The role of oligodendrocytes in immunomodulation. In recent years, there has been increasing evidence of the role of oligodendrocyte in inflammatory activation. OPC proliferation is induced by IGF-2, VEGF-A, and TGF-β, while the presence of IL-1β, IL-17, and IFNγ has an inhibitory function. Nevertheless, the presence of OPCs further induced the recruitment of peripheral immune cells, modulation of other glial cells, as well as some degree of phagocytic activity. Mature myelinating oligodendrocytes may act as APCs, by expressing specific receptors like MHC-I and -II, as well as TLR3 and TNFR2. Most importantly, Cx47 expressed at the soma and proximal processes of the oligodendrocyte support intercellular communication. IGF-2, insulin-like growth factor; VEGF-A, Vascular endothelial growth factor; TGFβ, Transforming growth factor beta; IL-1β, Interleukin 1-beta; IL-17, Interleukin-17; IFNγ, Interferon-gamma; CCL2, C-C motif chemokine ligand 2; FGF, Fibroblast growth factor; TNFR2, Tumor necrosis factor receptor 2; TLR3, Toll-like receptor 3; Cx47, Connexin 47; MHC, Major histocompatibility complex; T reg, B reg, T and B regulatory cells; TCR, T cell receptor (figure was created with BioRender.com).

Increasing evidence suggests that oligodendrocytes are actively involved in CNS immunomodulation (Figure 2) by expressing cytokines (IL-1b, IL-17A, CCL2), chemokines (C-X-C motif chemokine ligand, CXCL-1, CXCL10, CXCL12), APCs (MHC-I, II), complement and complement receptor molecules (C1, C1q, C3) and complement regulatory molecules (CD46, CD55) [72]. Thus, oligodendrocytes contribute to the inflammatory response in neurological diseases to preserve tissue homeostasis and neuronal integrity [76].

Oligodendrocytes were reported to express at least four chemokine receptors, CXCR1, CXCR2, CXCR3, and CXCR4 [77,78], which, upon binding with their respective ligands, are involved in the triggering of multiple signaling pathways. CXCL1 is upregulated in the peripheral areas of demyelination and, when recognized by CXCR2, prevents OPC migration in vivo [79]. Additionally, CXCR2 signaling protects oligodendrocytes from demyelination in a viral-induced mouse model of demyelination [80], while CXCR2-positive neutrophils are necessary for cuprizone-induced demyelination [81]. An important molecule in the migration, proliferation, and differentiation of neural precursor cells, CXCL12, is upregulated in the corpus callosum of mice after cuprizone-induced demyelination, along with increased astrocyte and microglia activation [82]. CXCR4 is a receptor for CXCL12 signaling, and the loss of CXCR4 signaling leads to decreased OPC maturation and remyelination blockage. The CXCL12-mediated migration of OPCs is facilitated by the CXCR4-activated mitogen-activated protein/extracellular signal-regulated kinase (MEK/ERK) and phosphatidylinositol 3-kinase/Akt (PI3K/AKT) pathways [83]. The inhibition of the mitogen-activated protein kinase (MAPK/ERK) pathway promotes oligodendrocyte differentiation and recovery from demyelination [84]. Additionally, cuprizone-induced demyelination leads to high levels of CCL2, which is critical for monocyte recruitment, and IL-1β, which is involved in many immune-mediated responses [85].

In order to interact with the neighboring immune cells, oligodendrocytes secrete a broad range of cytokines. They express IL-17A, a pro-inflammatory cytokine involved in the pathogenesis of autoimmune diseases [86], with a subpopulation expressing IL-17 in active lesions [87]. *IL-17*^−/−^ mice showed milder EAE pathogenesis compared to the WT, while the IL-17 neutralizing antibody administration partially relieves symptoms [88]. IL-4 and IL-10 presence in primary oligodendrocyte cultures led to reduced OPC differentiation and immune responses, while exposure to TNFα led to increased phagocytic activity, cytokine production, and MHC-II expression [89]. Additionally, oligodendrocytes and OPCs express MHC-II molecules in response to IFNγ, which regulated T cell survival and proliferation and activated CD4+ T cell memory and effect [67]. Additionally, OPCs exposed to IFNγ can act as antigen-presenting cells to cytotoxic CD8+ T cells in vivo and in vitro, leading to cytotoxic death [67,90]. Contrary to this, intrathecal and peripheral administration of IFNγ ameliorated EAE pathology, while IFNγ ablation in mice increased EAE susceptibility [91].

Additionally, the disruption of the BBB in MS is present also upon interaction of the vasculature with oligodendroglia, which triggers CNS inflammation by pronounced microglia/macrophage activation [65]. Adhesion molecules like intercellular adhesion molecule 1 (ICAM-1) and vascular cell adhesion protein (VCAM-1) are upregulated upon EAE induction, while blocking ICAM-1 could reduce the number of Th1 binding with oligodendrocytes in vitro, suggesting the interaction of the adhesion molecules with the CD4+ T cells [92]. Kv1.4 was previously identified to be involved in controlling OPC proliferation in vitro [93] and in vivo [94] and may interact with cells of the immune system, as blocking Kv1.4 expression exerts beneficial results in the acute EAE model but not in the cuprizone.

Oligodendrocyte and OPC survival, proliferation, and differentiation depends on the inflammatory microenvironment. Presence of effector T cells, IFNγ, and ROS affects the fate of OPCs by inhibiting differentiation and therefore remyelination [61]. The presence of inhibitory molecules in the inflammatory microenvironment of OPCs, like Lingo-1 that inhibits axonal regeneration after spinal cord injury [95], may also affect their fate. Similarly, the absence of proliferating factors, such as IGF1, TGFβ, and integrins can limit remyelination. Fibroblast growth factor (FGF) signaling is likely to play a role in oligodendrocyte regeneration and myelin formation, as FGF2 and/or FGFR were upregulated in MS patients and mouse models of demyelination. Moreover, *FGF1/FGFR2*^−/−^ mice showed less efficient myelin recovery in chronic but not in acute cuprizone demyelination [96].

The proinflammatory phenotype of OPCs that promotes damage and blocks remyelination suggests that the suppression of these pathways may ameliorate tissue destruction and promote OPC proliferation and differentiation into myelin-producing cells [90]. Oligodendrocytes were shown to express tumor necrosis factor receptor 2 (TNFR2) in the early phase of EAE pathogenesis, while TNFR2 ablation leads to an earlier onset and increased motor dysfunction after EAE induction, with a higher infiltration of immune cells [97].

Complement activation is a key feature in MS plaques, as studies have revealed that NAWM plaques were positive from complement proteins (e.g., C3, C1q), activation products (e.g., C3b, C4d), and various regulators (e.g., factor H) [98,99]. MS-specific recombinant antibodies activate the classical complement pathway, leading to oligodendrocyte death and rapid demyelination [100]. In corroboration with this, the C5b–9 complex was shown to prevent oligodendrocyte cell death via regulating the PI3K/AKT pathway [101] and inhibiting caspase-8 activity [102].

Helper T cells that drive adaptive immunity also play a key role in MS and the EAE animal model. Th17 polarization induces cytotoxic inflammation and inhibits remyelination in vitro [103]. Oligodendrocytes have close contact with Th17 cells upon EAE induction, which may worsen the pathology and increase cell death, possibly by the release of cell stress-inducing glutamate due to the increased expression of integrin CD29 [104].

### 2.2. Oligodendrocyte and Remyelination

The process of remyelination depends on a time-tight activation and recruitment of OPCs to the lesion site, followed by proliferation, differentiation, and maturation into myelinating cells, which contribute to the regeneration process. OPCs attempt to re-establish the myelin sheath of previously demyelinated axons [105] by coming into close contact with the denuded axon, expressing myelin-specific genes, and finally forming the new myelin sheath [106]. Remyelination is mostly found in acute and RRMS active lesions and borders [107], whereas mixed active/inactive lesions show a decrease in or absence of remyelination. Early remyelinated lesions are characterized by increased numbers of OPCs distributed in increasing gradient from the core of the lesion towards the periphery [95,108,109]. Pre-existing mature oligodendrocytes are able to generate further myelin sheaths; however, their remyelination capacity is limited [110], with adult OPCs being the main cell population responsible for the re-establishment of myelinated healthy axons. Mature oligodendrocytes at the lesion borders limit demyelination and favor myelin repair [111]. Interestingly, Schwann cells were shown to mediate remyelination within astrocyte-deficient areas where some of these also derive from OPCs in a transdifferentiation process [112]. However, OPC differentiation and efficient remyelination ultimately depends on the resolution of local inflammation [113].

Demyelination induces the secretion of various chemoattractants from astrocytes and microglia to activate OPC recruitment, survival, migration, proliferation, and differentiation to lesion sites, including IGF1, FGF2, IL-1β, TNFα, CXCL12, and platelet-derived growth factor (PDGF)-AA [114]. OPCs express functional receptors for cytokines like IL-10, IL-6, IFNγ, and others, which have a predominantly negative impact on their maturation and proliferation [115]. Migration and differentiation of OPCs is promoted by CCL2 and IL1β in vitro and in the cuprizone-induced demyelination model [85]. Additional mechanisms including the PDGF-A-induced ERK pathway promotes OPC migration in vitro [116], as well as CXCL12 stimulation of the MEK/ERK and PI3K/AKT pathways [83]. On the other hand, glia cells release a wide range of chemorepellents, such as ECM-remodeling enzymes, that inhibit the remyelination process by impairing OPC recruitment and enhancing the inflammatory process [117]. Furthermore, the Wnt signaling [118] and Notch pathways [119] inhibit OPC differentiation.

A potent contributor to OPC recruitment failure is the proinflammatory cytokine, IFNγ. IFNγ promotes the senescence of OPCs by upregulating the transcription factor paired related homeobox protein 1 (PRRX1), which prevents the proliferation and tissue colonization of transplanted human OPCs, and upon LPC-induced demyelination [120]. Moreover, IFNγ was shown to induce MHC-I expression on OPCs [121], which inhibits FGF receptor signaling and the proliferation of neural progenitor cells [122]. Aging WM OPCs responds to IFNγ, induced by CD8+ T cells, leading to oligodendrocyte loss [123]. Consequently, IFNγ signaling contributes to the failure of OPC proliferation at lesion areas and could be a potent target for remyelination-promoting therapies [124].

While myelin-reactive T cells are the key cells releasing chemicals and causing inflammation in the MS lesions, T cells were also shown to target OPCs and inhibit remyelination as well as CD8+ T cell infiltration into the CNS via the IFNγ-mediated promotion of antigen cross-presentation in the cuprizone-induced demyelination model [90]. Additionally, a hybrid model of the demyelination model, which combines cuprizone-induced demyelination with the adoptive transfer of myelin-specific CD4+ T cells, showed that infiltration of Th17 cells into the CNS correlates with impaired remyelination [125]. In contrast, T cell activation was shown to induce the proliferation of OPCs via releasing vascular endothelial cell growth factor-A (VEGF-A) [126] as well as the Treg-mediated release of cellular communication network 3 (CCN3), a growth regulatory protein [127].

The unmet need for the development of approaches aimed at regeneration and repair has led several groups to attempt to enhance OPC proliferation via the administration of various compounds. One such approach was adopted by Mei et al. [128], which screened for the efficiency of 1000 bioactive molecules and identified eight U.S. Food and Drug Administration-approved antimuscarinic compounds that enhanced oligodendrocyte differentiation and membrane wrapping. Additionally, the direct antagonism of the M1 and/or M3 muscarinic receptors by the drug benztropine enhanced remyelination in the cuprizone-induced demyelination model as well as EAE adoptive transfer by stimulating the differentiation of progenitor cells [129]. Moreover, the single-cell transcriptomic analysis of differentiating OPCs treated with various compounds identified two promising bioactive molecules, namely Ro1138452 and SR2211, that promote myelination in human pluripotent stem cells in vitro [130], providing evidence for the possible development of oligodendrocyte differentiation-targeting-based therapies.

## 3. Astrocytes and Neuroinflammation

**Figure 3:**
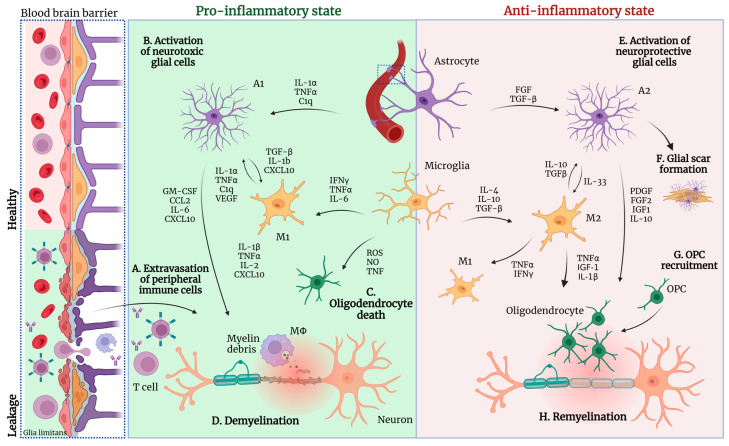
Glial cells in the center of inflammation and neurodegeneration in MS. During early MS, there is a significant compromise of the BBB, through which activated immune cells extravasate from the periphery into the CNS. Both astrocytes and microglia are activated into A1 and M1 states, respectively, and by secreting various pro-inflammatory molecules induce further in myelin destruction. Additionally, peripheral macrophages and microglia assist in myelin debris clearance. The activation of this proinflammatory state is devastating and determines the lesion formation. Later, both astrocytes and microglia shift their activation state into an anti-inflammatory, namely A2 and M2, respectively, whereby inducing scar formation and OPC recruitment, facilitate in myelin repair. Occasionally, there is activation back to the pro-inflammatory states, depending on environmental signals. IL-1α, Interleukin-1alpha; TNFα, Tumor necrosis factor alpha; C1q, Complement 1q; TGF-β, transforming growth factor-beta; IL-1β, Interleukin-1beta; CXCL10, C-X-C motif ligand 1; VEGF, Vascular endothelial growth factor; GM-CSF, Granulocyte-macrophage colony-stimulating factor; CCL2, C-C motif ligand 2; IL-6, Interleukin 6; IL-2, Interleukin 2; MΦ, Macrophage; ROS, Reactive oxygen species; NO, Nitric oxide; TNF, Tumor necrosis factor; IFNγ, Interferon gamma; FGF, Fibroblast growth factor; IL-10, Interleukin 10, IL-4, Interleukin-4; IGF-1, Insulin-like growth factor 1; IL-1β, Interleukin 1 beta; PDGF, Platelet derived growth factor; OPC, Oligodendrocyte precursor cell; IL-33, Interleukin 33 (figure was created with BioRender.com).

Astrocytes, accounting for approximately 40–50% of all glial cells in the CNS, form star-like processes. They play a key role in efficient interactions with other glial cells to maintain homeostasis and provide support to surrounding cells. Astrocytes promote myelination by facilitating OPC proliferation, differentiation, and mediating the oligodendrocyte–axon initial contact. Additionally, astrocyte end-feet surround blood vessels where they contribute to the formation of the BBB and create the glia limitans (Figure 3). There, they assist in the blood flow regulation and nutrient absorption through the water channel aquaporin 4 (AQP4) [131,132], potassium (K^+^) channels [133], and Cx43, which are expressed on the astrocyte end-feet. Furthermore, by taking up glucose from the bloodstream, they supply neurons with crucial energy substrates. They also play a key role in the synapses as they ensheathe them with their fine processes and participate in ion regulation, neurotransmitter release and osmotic gradient, as well as promote synaptogenesis and modulate synaptic pruning by labeling the synapses for microglia-mediated elimination [134]. Finally, they are the cells responsible for scar formation upon injury, known as astrogliosis, which prevents inflammation from spreading to the surrounding tissue; furthermore, while less than microglia, astrocytes have a phagocytic capacity [135,136].

Astrocytes arise from neural progenitor cells (NPCs) in the subventricular zone (SVZ) during development and migrate along radial glial processes to populate the brain [137]. Ramón y Cajal initially classified astrocytes into protoplasmic and fibrous, depending on their morphology, location and function as well as antigen phenotype [138]. Type 1 astrocytes (A1), or protoplasmic, are found in the GM and ensheathe synapses as well as blood vessels, promoting BBB functions, while type 2 astrocytes (A2), or fibrous, are found in the white matter, in close contact with the nodes of Ranvier and blood vessels. In addition to their classification by morphology, they are also divided into inactive (quiescent), active, and reactive. Inactive astrocytes are present in the normal resting tissue and become activated by various mechanisms, leading to various pro-inflammatory molecules release [139].

### 3.1. Astrocyte Dysregulation in MS

The activation of proinflammatory astrocytes leads to loss of their physiological functions and the secretion of cytokines and chemokines that regulate different events in MS development and progression, driving rapid neuronal and oligodendrocytic death [140]. Additionally, near the lesions, they present with morphological changes in respect to length and complexity, indicating metabolic changes [141]. Reactive astrocytes extend beyond the lesions, suggesting their contribution to lesion development. Moreover, the formation of glial scars, while detrimental in limiting further inflammation, may interfere with the ability of oligodendrocytes to facilitate remyelination. Therefore, understanding the contribution of astrocytes in different stages of MS lesion formation may prove crucial in developing possible strategies for intervention.

Astrocyte activity is initiated and modulated by various signaling pathways, including the nuclear factor kappa-light-chain-enhancer transcription factor (NFκB) [142], Janus kinase/signal transducer–activator of transcription factor 3 (JAK/STAT3) [143], and MAPK [144] pathways. NFκB nuclear translocation is triggered by the presence of TNFα, IL-1β, IL-17, and myelin debris. It contributes to the progression of EAE [145], while selective blockage improves its severity by decreased production of pro-inflammatory cytokines and oxidative stress [146,147,148].

Astrocytes express MHC molecules, which gives them a non-traditional APC phenotype [149]. Additionally, they express a wide range of immune receptors including pattern-recognition receptors (PRRs), which detect pathogen-associated molecular patterns (PAMPs), and tissue damage-associated molecular patterns (DAMPs). One major PAMP is the Toll-like receptor (TLR)-3, which is activated by double-stranded RNA, usually found in viral genome replication, and promotes neuroinflammation by the upregulation of CCL2, IL-1β, and CXCL10 [150]. TLR3 and TLR4 activation, shown to be upregulated in EAE as well as the blood cells of MS patients [151], suggests an inflammatory activation through NFκB and TNFα [149]. Conversely, TLR-3-mediated astrocyte activation induces an anti-inflammatory cytokine release, including IL-9 and IL-10, suggesting a modulatory role in MS patients [152].

Depending on the environment, anti- or pro-inflammatory stimulating molecules are secreted, which affect the de- and remyelination processes. The stimulation of the astrocytes upregulates or induces the secretion of cytokines like TNFα, IL-1β, brain-derived neurotrophic factor (BDNF), VEGF, as well as chemokines including CCL2 and CXCL10 [37]. Cell-specific and region-specific transcriptomics showed increased expression of immune pathways related to astrocyte function in the EAE mouse model [153]. Moreover, persistent activation of astrocytes alters astrocytic cell signaling, protecting oligodendrocyte degeneration from cuprizone-induced demyelination via reduced CXCL10-mediated NFκB signaling [154]. These studies corroborate with the current theory that astrocytes can have a detrimental effect in the demyelination process by inducing further inflammation as well as promote its resolution when given the appropriate signals.

When astrocytes forming the glia limitans become activated, they may alter the environment of the BBB and increase permeability [155]. In MS patients, the perivascular astrocytic end-feet formation was shown to be damaged in early lesions, indicating the possibility that astrocyte lesions may drive demyelination [156,157]. Additionally, astrocytes facilitate the T cell entry into the CNS via expression of VCAM-1, as shown in the EAE mouse model [158]. Interestingly, AQP4 was upregulated in both the postmortem MS tissue [159] and EAE mouse model [160], while the ablation of AQP4 ameliorates EAE progression [161]. Moreover, astrocyte activation leads to increased CCL2 production, which plays a key role in the recruitment of immune cells during chronic EAE [156,157,162].

While astrocytes play a key role in the compromising of the BBB, they also release chemokines that favor the recruitment of circulating leukocytes [163] and the induction of differentiation of CD4+ T cells into a pro-inflammatory state, as well as CD8+ T cell cytotoxic activity [164]. Th1- and Th17-derived factors induce a proinflammatory phenotype in astrocytes [165], with an increased expression of IL-1β, IL-6 and NOS2, as well as chemokines CCL2, CCL20, CXCL10 [166]. Additionally, during EAE, Th1, and Th17 induced the production of GM-CSF [167], which mediates neuroinflammation by activating microglia, BBB disruption by enhancing expression of adhesion molecules like ICAM-1 and VCAM-1, as well as tight junction disassembly via zonula occludens 1 (ZO-1) transcription downregulation [168]. Th1-mediated release of IFNγ triggered the microglia to release IL-1β, which in turn downregulated Cx43 in astrocytes, disrupting the astrocytic intercellular communication [169].

Additionally, a postmortem MS brain showed elevated IL-27 levels and its cognate receptor (IL-27R) [170], which impacted the regulation of immune gene expression, including CXCL9, CXCL10, CXCL11, as well as programmed death-ligand 1 (PD-L1), human leukocyte antigen (HLA)-E, and ICAM-1 [171]. Additionally, CXCL10 produced by the activated astrocytes stimulated microglia recruitment in the cuprizone model [172], and the selective ablation of reactive astrocytes in vivo ameliorated EAE progression by decreasing microglial activation and monocyte infiltration [173].

The role of the complement has emerged as a crucial component in the controlling of immunological responses, as the CNS actively produces components of innate immunity, like complement proteins [174]. C1q and C3 components determine the cellular and humoral immune responses in the progression of MS, participating in astrocytosis, microgliosis, and synaptic engulfment. Reactive astrocytes express the C5a complement receptor [175,176] as well as C1q and C3 [177]. Synaptopathy occurs in MS, independent of demyelination, and further expands the disease progression. The induction of EAE leads to the increased bioavailability of C1q and C3, which modulates glutamate release from astrocytic compartments, while affecting the activity at nerve terminals and impairing astrocytic processes [178].

### 3.2. The Role of Astrocytes in Remyelination

The transition from demyelination to remyelination leads to a shift in activation status of astrocytes. Transcriptomic analysis of the astrocytes after cuprizone-induced demyelination and upon short and longer withdrawal, where remyelination takes place, showed that they underwent significant transcriptional changes in each phase [179]. This large spectrum of astrocyte functions contributes to both disease progression and repair.

After CNS injury, astrocytes secrete inhibitory molecules and extracellular matrix components, which are detrimental to the remyelination process [180]. The expression of endothelin-1 (ET-1) was shown to inhibit remyelination via the Notch pathway [181], while ET-1 signaling caused an upregulation of the Notch1 receptor ligand, jagged-1, inhibiting the interaction between astrocytes and oligodendrocytes [182]. Remyelination is also impaired due to the glial scar formation and astrocyte production of inhibitory molecules, PDGF [183] and FGF2 [184]. Additionally, while the depletion of Cx43 did not affect LPC-induced demyelination, it accelerated remyelination, indicating that Cx43 hemichannels may regulate negatively the remyelination process by favoring local inflammation [54].

Recent evidence, however, supports a crucial role of astrocytes in remyelination, exerting various functions, such as myelin debris removal, to induce faster and more effective remyelination [185]. Reactive astrocytes present at the lesion edges, express chemoattractant molecules for OPCs, including CXCL1, CXCL8, and CXCL10, inducing their migration toward the demyelinated lesion. Additionally, astrocytes promote OPC proliferation by secreting PDGF, FGF2, leukemia inhibitory factor (LIF), ciliary neurotrophic factor (CNTF), and IGF-1 [186]. Furthermore, astrocytes support the survival of regenerating oligodendrocytes via the upregulation of cholesterol synthesis/export through the Nrf2 pathway [35]. Moreover, astrocytes induce the secretion of exosomes by OPCs via Cx47 channels, which contain laminin subunit beta-2 (LAMB2) and therefore accelerate OPC proliferation [187] and increase the number of sphingosine-1-phosphate receptors 3 (Sp1r3) on OPCs via Cx47-mediated direct contact, further promoting their proliferation [188].

As remyelination is more efficient in patients of a young age, it is presumed that astrocyte activation is beneficial through the secretion of both permissive and inhibitory molecules. Later, the balance between pro- or anti-inflammatory state is lost, and astrocytes become more toxic rather than regenerative [180]. Nevertheless, whether the effects of astrocytes are beneficial or detrimental is much more complex, as it depends on a temporal dynamic interplay between all the cells involved in the process.

Astrocytes are thought to be promising therapeutic targets for MS management, due to their ability to communicate with oligodendrocytes and support myelin formation. Although many studies are focusing on promoting remyelination via OPC differentiation-related pathways, one should consider the microenvironment in which these cells need to survive and function. Understanding astrocyte diversity as well as crosstalk with other glia cells is crucial to develop astrocyte-mediated therapeutic strategies [189].

## 4. Microglia and Neuroinflammation

The microglial cell population, comprising around 5–20% of the total glial population, depending on health state, are the resident macrophages of the CNS. By being members of the innate immune response, they constantly survey their microenvironment for tissue damage [190] and are actively involved in antigen presentation. They also play important roles in myelin development, preservation, and growth, while the absence of microglia leads to altered lipid metabolism in oligodendrocytes through disrupted TGFβ function [191]. Microglia mediate the clearance of myelin debris and misfolded proteins in response to injury by phagocytosis, which facilitates the efficient remyelination and recruitment of OPCs [192]. They are also involved in promoting synaptic plasticity [193,194,195] as well as neuronal function by maintaining continuous interactions with neighboring cells [56]. Chronic activation of microglia during neurodegeneration leads to the prolonged release of proinflammatory molecules, which can be both detrimental and protective depending on the microenvironment [11].

Microglia are highly dynamic cells and, at resting state, they are ramified cells with small somata and multiple processes, which protrude and retract in order to survey large brain areas [196]. When activated, they gain an amoeboid morphology which allows for a proinflammatory function, as they exert shorter ramifications and have larger cell somata [197]. Phagocytic microglia have no ramifications but have large somata, while dystrophic microglia are considered to be present in the aging brain [198].

Depending on their state of activation, they either induce nerve growth or neurotoxicity [199]. Microglia express a distinct profile including TGFβ [200], interferon regulatory factor 8 (IRF8), and transmembrane protein 119 (TMEM119) [201]. Like astrocytes, microglia express PRRs, including TLRs and NOD-like receptor (NLRs), that recognize PAMPs and DAMPs [202] and therefore control inflammation. Additionally, they are able to sense inflammatory mediators via chemokine and cytokine receptors, like TNFα, IFNs, TGFβ1, and ILs [56].

Microglia are usually categorized as classically stimulated proinflammatory (M1) or alternatively activated anti-inflammatory cells (M2), depending on their polarization state. M1 microglia are stimulated by IFNγ and secrete proinflammatory cytokines IL-1β, IL-6 and TNFα [139] as well as C1q [140], that contribute to the inflammatory response. Conversely, polarization to M2 by cytokines IL-4 or IL-3 leads to the secretion of the anti-inflammatory and neurotrophic factors IL-10, IGF1, and TGFβ [203]. Advances in technologies such as mass cytometry and single-cell RNA sequencing (sc-RNA seq) have allowed a deeper understanding of the microglia phenotype, both in healthy as well as in MS brains, offering new insights into the potential immune mechanisms [196,204,205].

### 4.1. The Role of Microglia in MS

There is emerging evidence that implicates microglia in MS pathology, directly or indirectly. The continuous activation of microglia drives neuroinflammation and neurodegeneration, while microglia nodules are associated with more severe MS pathology [206]. Moreover, microglial depletion prevents demyelination, oligodendroglial loss, and reactive astrocytosis upon cuprizone-induced demyelination [207]. Additionally, blocking mitochondrial complex-I in proinflammatory microglia limits neurotoxic damage and improves functionality in the EAE model [208]. Hence, the modulation of microglial activation comprises a promising target for MS management.

In early lesion formation, there is increased pro-inflammatory microglia accumulation [14] that later changes into mixed pro- and anti-inflammatory microglia [209,210] (Figure 3). Microglia express TMEM119 at the initial stage of new lesion formation, indicating that resident microglia dominate the lesion site, while their number drops when the lesion matures, and the recruitment of monocytes and other peripheral cells takes place [211]. Additionally, microglia expressing MHC-II are initially present, which serve as APCs. Increased microglia numbers are also present in the NAWM of MS patients compared to controls, especially in progressive MS [212]. In inactive lesions, microglia density is significantly reduced, while in chronic active lesions microglia, they form a rim surrounding the lesion [213], which is rich in iron and myelin debris. Chronic inactive lesions, however, are devoid of microglia/macrophages. While patterns of microglia activation are similar in different pathological conditions including stroke lesions, MS is dominated by a chronic microglia activation, which is thought to drive lesion transformation.

Microglial activation plays a fundamental role in MS lesions and disease progression where they exert both beneficial and detrimental effects. During demyelination, microglia clear the myelin debris by phagocytosis and secrete proinflammatory cytokines IL-1β, IL-6, IL-12, IL-13, IL-18, IFNγ, and TNFα, and chemokines including CCL2, CCL3, CCL4, CCL5, and CCL12 [214]. CCL5 promotes the activation of MMP-9, which is involved in leukocyte extravasation as well as their link with myelin degradation products [201,215].

CX3CL1, also known as fractalkine, is a transmembrane protein produced by neurons that interacts through its receptor CX3CR1, which is located on microglia [216]. *CX3CR1*^−/−^ show decreased phagocytosis by microglia, and the persistence of myelin debris that inhibits proper remyelination due to impaired OPC recruitment [217]. CX3CR1+ microglia exert protective effects by engulfing and destroying Th17 cells upon EAE induction [218], which are predominantly present in the lesions and serum of MS patients [219] and are considered critical regulators of the disease course [220].

Growing evidence indicates that microglia colocalize with T cells in MS lesions [221], and their presence correlates with axonal damage. Interactions between T cells and microglia may be present not only via soluble factors but also via direct cell-to-cell contact [222]. Microglia secrete IL-12 and IL-13, which promote Th1 accumulation [223]. In turn, Th1 cells activate further resident microglia into the M1 phenotype [224] and upregulate expression of MHC and other costimulatory molecules, further favoring Th1 reactivation and infiltration [225]. Moreover, Th1- and Th17-induced astrocyte stimulation releases factors that enhance microglia migration in vitro [166].

As previously mentioned, microglia express various TLRs, including TLR2, TLR4, and TLR5. TLR2 was shown to be upregulated in demyelinating MS lesions [226], while TLR4 was increased in cerebrospinal fluid (CSF) mononuclear cells [227]. Increased expression of the latter suppresses the polarization of the M1 to M2 phenotype and therefore prolongs proinflammatory response [228]. TLR3, 5, 7, 8, and 9 are expressed by dendritic cells and have been shown to be present in a subset of MS patient lesions [229].

The transcription factor NFκB regulates various aspects of the immune functions of both the innate and adaptive immunity, including encoding cytokines and chemokines, as well as inflammasome activation like the leucine-rich repeat- (LRR-) and NOD-like receptor protein 3 (NLRP3) [230] by inducing pro-IL-1β [231]. It is activated by factors like IL-33, IL-1b, IL-12, GM-CSF, TNF-α, and IL-17. The conditional knockout of inhibitory kappa B kinase beta (Ikkβ), a key regulatory kinase in the activation of NF-κB, in mice conditioned with EAE, exhibited ameliorated progression as reduced levels of microglia infiltrated lesions decreased M1 polarization and CD4+ T cell response [232].

Colony-stimulating factor 1 receptor (CSF1R) is required for the proper development of microglia and macrophages and is a critical regulator of homeostasis. CSF1R was shown to be upregulated in microglia-associated diseases [233] as well as in MS [234]. Activated microglia drive demyelination through the CSF1R signaling [207], especially in progressive MS, as neuroinflammation persists with constant survival and proliferation of microglia [235], while CSF1R inhibition ameliorates neuroinflammation and microglial activation in the EAE mouse model of MS [234].

TNFα is a major regulator of inflammatory response, mainly secreted by activated microglia via extracellular vesicles as well as macrophages, T cells, and natural killer (NK) cells that mostly exert cell death signals. It drives microglia into M1 polarization, leading to further TNFα secretion [236]. TNFα is upregulated in EAE and cuprizone-induced demyelination models as well as MS lesions [237], upregulating the NFκB signaling pathway and enhancing the secretion of various pro-inflammatory molecules [238]. IL-9 was shown to modulate microglia inflammatory activity by reducing the expression of triggering receptors expressed on myeloid cells-2 (TREM-2) and TNF release in the EAE mouse model, improving clinical disability and mitigating synaptic damage [239]. However, even though anti-TNFα treatment resulted in the reduced incidence and delayed onset of EAE, no effect on disease severity was observed [240].

IFNγ release is considered a hallmark of Th1-driven inflammation in MS and EAE [241]. It can also trigger microglia to act as effector cells, causing damage via the release of cytotoxic factors [242] and, along with TLR4 coactivation, can result in massive dysfunction and cell death [243]. However, IFNγ was also shown to induce microglia apoptosis, possibly having a pivotal role in a self-limiting negative feedback mechanism, exerting a beneficial effect in the disease progression. Additionally, IFNγ administration ameliorated EAE progression via decreased CD11b+ myeloid cells and inflammatory cell infiltration [244].

Microglial contribution to MS pathology is a decisive factor in the disease progression. They have attracted increasing attention due to their diverse functions, from maintaining homeostasis and supporting neurons to actively favoring myelination. Extracellular vesicles secreted by microglia can influence their microenvironment, signaling astrocytes into proinflammatory states as well as promoting a pro-regenerative milieu [245]. Their versatile roles in the pathogenesis of neuroinflammation, neurodegeneration, and repair, are crucial in biomarker discovery, as well as in more targeted therapeutic interventions.

### 4.2. Microglia in Remyelination

Microglia are highly dynamic cells as changes in activation, transcription, and proteomics allow them to regulate their functions in a temporal- and context-dependent manner in response to de- and remyelination [246,247]. Although increasing evidence implicates microglia in the pathology that underpins neurological disruption in MS, microglia function can orchestrate the remyelination process. Microglia and macrophages are typically located at the lesion border, where the first signs of remyelination are present, and their profile can decide the fate of remyelination. Microglia of the M2 phenotype are present in high densities at areas of active remyelination and not where remyelination is impaired, highlighting the importance of transition of microglia into an anti-inflammatory type [248]. Corroborating this, recent evidence has shown that microglia polarization to the M2 phenotype regulates spontaneous remyelination after intermittent cuprizone-induced demyelination [249]. Much like astrocytes, microglia can create both a beneficial and a hostile environment in respect to tissue regeneration.

Microglia promote remyelination by the efficient phagocytosis of myelin debris, secretion of regenerative factors and regulation of the ECM [250], while persistent activation is associated with impaired remyelination. Even though microglia activation may drive demyelination, myelin debris clearance by phagocytic microglia is thought to be a key step in promoting a favorable environment for remyelination [41,250,251,252,253,254]. However, efficiency of myelin debris clearance declines with aging [255]. This complex process involves internalization of the myelin debris, lysosome maturation, and cholesterol recycling. Interestingly, microglia also produce the immediate cholesterol precursor, desmosterol, which aids the resolution of inflammation and facilitates efflux of lipid/cholesterol to oligodendrocytes, a critical requisite for myelin synthesis [256].

One key mediator of myelin debris clearance and breakdown is CX3CR1, which is highly expressed in microglia and macrophages. More specifically, CX3CR1 ablation leads to the phagocytes lacking myelin debris after demyelination, suggesting an impairment in the internalization process [217]. However, compensatory mechanisms are present, as even if less OPCs are present, the *CX3CR1*^−/−^ mice showed some degree of remyelination. Moreover, myelin debris uptake was shown to be regulated by TREM2, which is regulated by CSF1R signaling. The conditional knockdown of CSF1R led to impaired myelin clearance after cuprizone-induced demyelination and debris accumulation [257]. TLR4 signaling has also been associated with myelin debris clearance, as well as regulation of cytokine and growth factor expression, which directly affect OPC replacement and remyelination [258]. Moreover, TLR2 ablation enhanced remyelination in the LPC mouse model [226], while reduced TLR2 signaling was associated with improved myelin recovery [259].

Beyond creating a remyelination-friendly environment via phagocytosis, microglia can directly regulate OPC responses [260]. Initially, a proinflammatory phenotype is abundant, aiding in the recruitment of OPCs to lesions, which changes into an anti-inflammatory phenotype at the oligodendrocyte differentiation and remyelination phase [250]. Microglia express Semaphorin-3F, which is a chemoattractant of OPCs to the lesion site [261]. Moreover, the secretion of inflammation-induced factors regulates oligodendrocyte development, especially microglia-secreted TNFα [262], IGF1 [263], and IL-1β [264]. Microglia deletion reduces OPC differentiation, even in the presence of phagocytic monocyte-derived macrophages [265], indicating further mechanisms in microglia activation to support remyelination.

Overall, microglia display a heterogeneous profile in activation state depending on their microenvironment, which is strongly correlated with underlying pathological processes, making them potential therapeutic targets in MS. Remyelination-specific MHC-II-related molecules, including HLA-DRB1 and CD74, found in human postmortem lesions, indicate the immunoregulatory role of subclusters of microglia [266]. However, due to the various characteristics denoted in microglia, and the absence of specific markers and molecular signatures, a more detailed assessment of microglia activity is needed to understand disease progression and plan therapeutic interventions.

## 5. Glial Cell Targeting in MS Treatment

While multiple approved DMTs are currently used as safe and effective options in the treatment of MS, they mainly rely on the modulation of the inflammatory response but not on the disease progression or functional recovery. Additionally, depending on the drug response and adverse effects, one needs to consider the escalation, de-escalation, or switching of DMTs to balance the risks and benefits of each treatment [267]. Therefore, an unmet need for new therapeutic strategies has emerged, especially for the progressive phase of the disease. More specifically, it is imperative to create new therapies for patients with highly active disease, as they have an increased risk for future disability as well as therapies to slow down or prevent the disease and/or disability with more targeted delivery [268]. Consequently, exploring innovative treatments to target the autoimmune reaction more broadly may offer new prospects in addressing the complexity of the disease.

Given the lack of approved therapies targeting myelin maintenance or regeneration, the current therapeutic strategies focus on targeting oligodendrocyte differentiation and maturation to become myelinating cells. Induced pluripotent stem cells (iPSCs) might be used as precursors to mature myelinating oligodendrocytes and may not only address remyelination issues in MS but other dysmyelinating disorders as well [268]. Moreover, the stimulation of endogenous remyelination via the promotion of OPC proliferation and maturation has been addressed. Compounds showing promising results include the antibody rHIgM22 [269], the DNA aptamer LJM-3064 [270] and Nogo-related blocking antibodies. Though, thus far, studies have mainly utilized iPSCs-derived neurons, drug development may shift towards iPSC-derived glia, given their emerging potential.

Various studies have shown the involvement of glial cells in orchestrating the complex immune response during the course of MS. Both microglia and astrocytes contribute to myelin injury and axonal damage, making them good candidates for therapeutic targeting. As discussed in the previous sections, glial cells have pivotal roles in the inflammatory cascade, by regulating leukocyte trafficking, releasing neurotoxic and neuroprotective factors, as well as limiting inflammatory damage. These pathways provide additional molecular targets for pharmacological intervention, which need to target specifically the detrimental activity of glia cells while preserving their reparative functions.

Current MS therapies were shown not only to mediate the adaptive immune activity but also influence glial cell function via the modulation of astrocyte or microglia activity. While initially fingolimod effectiveness was attributed to the modulation of T cell populations, non-lymphocyte-related mechanisms were introduced, especially via the regulation of astrocytic activity [271]. More specifically, among others, the promotion of astrocyte migration via the sphingosine-1 protein-1 (S1P1) receptor [272] and mediation of ERK phosphorylation [273] were shown to contribute to the drug’s effectiveness. Moreover, dimethyl fumarate induces the expression of Nrf2 in astrocytes and therefore upregulation of oxidative stress-induced growth inhibitor (OSGIN1), which is thought to limit astrocyte-mediated damage and leakage of the BBB by preventing the retraction of the perivascular astrocytic feet [274]. As the modulation of astrocytic activity is a major target in developing therapeutic strategies, one should take into account the diverse astrocyte functions, including scar formation and neuroinflammation [18].

As microglia can contribute directly or indirectly to the inflammatory and remyelination processes, modulation of their activity may strongly influence MS progression. DMTs were shown to modulate microglia activity [213], with fingolimod showing the most direct effect on S1P receptors on microglia, leading to the downregulation of IL-6, IL-1β, and TNFα [275]. Dimethyl fumarate modulates microglia differentiation into an anti-inflammatory type, leading to the reduced secretion of pro-inflammatory cytokines via the NFκB pathway [276]. Additionally, glatiramer acetate exerts neuroprotection via the activation of proinflammatory M2 microglia [277]. Various other agents are being tested in a pre-clinical setting and, even though targeting microglia seems more plausible than astrocytes, at different stages of the disease, microglia exert different properties, therefore a deeper understanding and characterization of the distinctive pathways influenced by microglia activation may allow for the development of microglia-directed treatments.

A potential therapeutic effect may arise from the nanomodulation of glial cells as well as macrophages migrating to the CNS [278]. It has been suggested that macrophages may be used as carriers of therapeutic agents as, due to the limiting crossing of pharmacological agents though the BBB, they make great candidates for transferring cargo via a more natural chemokine-induced migration to the brain. Inflammation in the brain leads to upregulated secretion of ICAM-1 and therefore BBB breakdown. Macrophage-secreted exosomes were shown to interact with the BBB endothelial cells, leading to increased BBB leakage, which can be used as a means for more effective drug delivery [279]. Overall, utilizing macrophages as delivery systems of small drugs or RNAs may provide a new perspective in the management of inflammation in MS.

Nanoparticle delivery loaded with myelin antigens and other tolerogenic adjuvants induced antigen-specific tolerance, ameliorating chronic progressive EAE [280,281]. Additionally, mRNA vaccines coding for disease-related autoantigens showed disease suppression in the EAE mouse model [282]. Overall, these approaches target bystander immune system activation; however, it still remains unclear whether this is sufficient to limit the ongoing inflammation in MS.

A key reason in further understanding the inflammatory response in MS is the potential identification of disease biomarkers, facilitating the prediction of disease future course. Recent studies have proposed the use of profiling the cytokine and chemokine levels in the CSF and serum of MS patients, especially in RRMS [283], as dynamic biomarkers can indicate the disease severity and level of inflammatory activity [284]. Moreover, the use of microglia and/or astrocyte-secreted molecules and cytokines may have potential usage as markers for MS [214]. Recent studies have identified chitinase-3-like protein 1 (CHI3L1) to be a biomarker for MS progression, especially predicting disability progression in the serum [285], plasma [286], as well as in the CSF [287] of progressive MS patients. More specifically, CHI3L1 release was associated with both microglia/macrophage [288] and astrocytes [289,290], holding promise for targeted therapy of inflammatory demyelination. Finally, immunophenotyping MS patients before the initiation of treatment may serve as a powerful tool to offer more targeted, personalized treatment for each patient and disease course [291].

Given the significant role of glial cells in the pathogenesis and progression of MS, modulation of the detrimental neuroinflammatory process could potentially be a crucial milestone in disease management. However, glial cells function in a synergistic manner, and therefore glial–glial interactions have emerged as a potent target for promoting remyelination. As remyelination tightly depends on the involvement of multiple cell types around the lesion, understanding glial cell interaction may prove to be key in the pathway towards promoting efficient tissue repair.

## 6. Concluding Remarks and Future Perspectives

Over the years, increasing evidence has shown the involvement of glial cells in the underlying pathological mechanisms in MS. A milieu of stimuli can determine whether they exert pro- or anti-inflammatory effects and to what extent; however, a deeper understanding of these finely tuned effects of damage and repair shall prove detrimental in developing effective therapies for immune-driven diseases like MS. The proper function of the CNS relies on the communication of glial cells, and understanding these complex dynamic interactions will allow for more specialized targeting when considering potential therapeutic targets that can be combined with the immune intervention of myelin repair.

## References

[1] Composton A., Coles A. (2008). Multiple Sclerosis. Lancet.

[2] Filippi M., Rocca M.A. (2011). MR imaging of multiple sclerosis. Radiology.

[3] Brosnan C.F., Battistini L., Raine C.S., Dickson D.W., Casadevall A., Lee S.C. (1994). Reactive nitrogen intermediates in human neuropathology: An overview. Dev. Neurosci..

[4] Brambilla R., Morton P.D., Ashbaugh J.J., Karmally S., Lambertsen K.L., Bethea J.R. (2014). Astrocytes play a key role in EAE pathophysiology by orchestrating in the CNS the inflammatory response of resident and peripheral immune cells and by suppressing remyelination. Glia.

[5] Szychowski K.A., Skora B., Wojtowicz A.K. (2022). Elastin-Derived Peptides in the Central Nervous System: Friend or Foe. Cell. Mol. Neurobiol..

[6] Ma J., Wang B., Wei X., Tian M., Bao X., Zhang Y., Qi H., Zhang Y., Hu M. (2024). Accumulation of extracellular elastin-derived peptides disturbed neuronal morphology and neuron-microglia crosstalk in aged brain. J. Neurochem..

[7] Pisa M., Watson J.L., Spencer J.I., Niblett G., Mahjoub Y., Lockhart A., Yates R.L., Yee S.A., Hadley G., Ruiz J. (2024). A role for vessel-associated extracellular matrix proteins in multiple sclerosis pathology. Brain Pathol..

[8] Durelli L., Conti L., Clerico M., Boselli D., Contessa G., Ripellino P., Ferrero B., Eid P., Novelli F. (2009). T-helper 17 cells expand in multiple sclerosis and are inhibited by interferon-beta. Ann. Neurol..

[9] Dalakas M.C. (2008). B cells as therapeutic targets in autoimmune neurological disorders. Nat. Clin. Pract. Neurol..

[10] Magliozzi R., Howell O., Vora A., Serafini B., Nicholas R., Puopolo M., Reynolds R., Aloisi F. (2007). Meningeal B-cell follicles in secondary progressive multiple sclerosis associate with early onset of disease and severe cortical pathology. Brain.

[11] Muzio L., Viotti A., Martino G. (2021). Microglia in Neuroinflammation and Neurodegeneration: From Understanding to Therapy. Front. Neurosci..

[12] Trobisch T., Zulji A., Stevens N.A., Schwarz S., Wischnewski S., Ozturk M., Perales-Paton J., Haeussler M., Saez-Rodriguez J., Velmeshev D. (2022). Cross-regional homeostatic and reactive glial signatures in multiple sclerosis. Acta Neuropathol..

[13] Sen M.K., Mahns D.A., Coorssen J.R., Shortland P.J. (2022). The roles of microglia and astrocytes in phagocytosis and myelination: Insights from the cuprizone model of multiple sclerosis. Glia.

[14] Prineas J.W., Lee S. (2023). Microglia subtypes in acute, subacute, and chronic multiple sclerosis. J. Neuropathol. Exp. Neurol..

[15] Ponath G., Park C., Pitt D. (2018). The Role of Astrocytes in Multiple Sclerosis. Front. Immunol..

[16] Ponath G., Ramanan S., Mubarak M., Housley W., Lee S., Sahinkaya F.R., Vortmeyer A., Raine C.S., Pitt D. (2017). Myelin phagocytosis by astrocytes after myelin damage promotes lesion pathology. Brain.

[17] Frohman E.M., Racke M.K., Raine C.S. (2006). Multiple sclerosis—The plaque and its pathogenesis. N. Engl. J. Med..

[18] Healy L.M., Stratton J.A., Kuhlmann T., Antel J. (2022). The role of glial cells in multiple sclerosis disease progression. Nat. Rev. Neurol..

[19] Boyd A., Zhang H., Williams A. (2013). Insufficient OPC migration into demyelinated lesions is a cause of poor remyelination in MS and mouse models. Acta Neuropathol..

[20] Sun R., Jiang H. (2024). Border-associated macrophages in the central nervous system. J. Neuroinflammation.

[21] Chu T., Shields L.B.E., Zeng W., Zhang Y.P., Wang Y., Barnes G.N., Shields C.B., Cai J. (2021). Dynamic glial response and crosstalk in demyelination-remyelination and neurodegeneration processes. Neural Regen. Res..

[22] Cunniffe N., Coles A. (2021). Promoting remyelination in multiple sclerosis. J. Neurol..

[23] Constantinescu C.S., Farooqi N., O’Brien K., Gran B. (2011). Experimental autoimmune encephalomyelitis (EAE) as a model for multiple sclerosis (MS). Br. J. Pharmacol..

[24] Bjelobaba I., Begovic-Kupresanin V., Pekovic S., Lavrnja I. (2018). Animal models of multiple sclerosis: Focus on experimental autoimmune encephalomyelitis. J. Neurosci. Res..

[25] Franklin R.J., Gallo V. (2014). The translational biology of remyelination: Past, present, and future. Glia.

[26] Maciak K., Dziedzic A., Saluk J. (2023). Remyelination in multiple sclerosis from the miRNA perspective. Front. Mol. Neurosci..

[27] Chari D.M. (2007). Remyelination in multiple sclerosis. Int. Rev. Neurobiol..

[28] Parrilla G.E., Gupta V., Wall R.V., Salkar A., Basavarajappa D., Mirzaei M., Chitranshi N., Graham S.L., You Y. (2024). The role of myelin in neurodegeneration: Implications for drug targets and neuroprotection strategies. Rev. Neurosci..

[29] Medzhitov R. (2021). The spectrum of inflammatory responses. Science.

[30] Magni G., Riboldi B., Ceruti S. (2024). Human Glial Cells as Innovative Targets for the Therapy of Central Nervous System Pathologies. Cells.

[31] Ahmad S., Srivastava R.K., Singh P., Naik U.P., Srivastava A.K. (2022). Role of Extracellular Vesicles in Glia-Neuron Intercellular Communication. Front. Mol. Neurosci..

[32] Wilkins A., Majed H., Layfield R., Compston A., Chandran S. (2003). Oligodendrocytes promote neuronal survival and axonal length by distinct intracellular mechanisms: A novel role for oligodendrocyte-derived glial cell line-derived neurotrophic factor. J. Neurosci. Off. J. Soc. Neurosci..

[33] Howell O.W., Rundle J.L., Garg A., Komada M., Brophy P.J., Reynolds R. (2010). Activated microglia mediate axoglial disruption that contributes to axonal injury in multiple sclerosis. J. Neuropathol. Exp. Neurol..

[34] Fields R.D., Stevens-Graham B. (2002). New insights into neuron-glia communication. Science.

[35] Molina-Gonzalez I., Holloway R.K., Jiwaji Z., Dando O., Kent S.A., Emelianova K., Lloyd A.F., Forbes L.H., Mahmood A., Skripuletz T. (2023). Astrocyte-oligodendrocyte interaction regulates central nervous system regeneration. Nat. Commun..

[36] Matejuk A., Ransohoff R.M. (2020). Crosstalk Between Astrocytes and Microglia: An Overview. Front. Immunol..

[37] Linnerbauer M., Wheeler M.A., Quintana F.J. (2020). Astrocyte Crosstalk in CNS Inflammation. Neuron.

[38] Kumar Jha M., Jo M., Kim J.H., Suk K. (2018). Microglia-Astrocyte Crosstalk: An Intimate Molecular Conversation. Neurosci..

[39] Rothhammer V., Borucki D.M., Tjon E.C., Takenaka M.C., Chao C.C., Ardura-Fabregat A., de Lima K.A., Gutierrez-Vazquez C., Hewson P., Staszewski O. (2018). Microglial control of astrocytes in response to microbial metabolites. Nature.

[40] Peferoen L., Kipp M., van der Valk P., van Noort J.M., Amor S. (2014). Oligodendrocyte-microglia cross-talk in the central nervous system. Immunology.

[41] Lombardi M., Parolisi R., Scaroni F., Bonfanti E., Gualerzi A., Gabrielli M., Kerlero de Rosbo N., Uccelli A., Giussani P., Viani P. (2019). Detrimental and protective action of microglial extracellular vesicles on myelin lesions: Astrocyte involvement in remyelination failure. Acta Neuropathol..

[42] Kleopa K.A., Orthmann-Murphy J., Sargiannidou I. (2010). Gap Junction Disorders of Myelinating Cells. Rev. Neurosci..

[43] Kleopa K.A., Orthmann J.L., Enriquez A., Paul D.L., Scherer S.S. (2004). Unique distributions of the gap junction proteins connexin29, connexin32, and connexin47 in oligodendrocytes. Glia.

[44] Nagy J.I., Ionescu A.V., Lynn B.D., Rash J.E. (2003). Coupling of astrocyte connexins Cx26, Cx30, Cx43 to oligodendrocyte Cx29, Cx32, Cx47: Implications from normal and connexin32 knockout mice. Glia.

[45] Menichella D.M., Goodenough D.A., Sirkowski E., Scherer S.S., Paul D.L. (2003). Connexins Are Critical for Normal Myelination in the CNS. J. Neurosci..

[46] Odermatt B., Wellershaus K., Wallraff A., Seifert G., Degen J., Euwens C., Fuss B., Bussow H., Schilling K., Steinjauser C. (2003). Connexin 47 (Cx47)-Deficient Mice with Enhanced Green Fluorescent Protein Reporter Gene Reveal Predominant Oligodendrocytic Expression of Cx47 and Display Vacuolized Myelin in the CNS. J. Neurosci..

[47] Basu R., Das Sarma J. (2018). Connexin 43/47 channels are important for astrocyte/oligodendrocyte cross-talk in myelination and demyelination. J. Biosci..

[48] Kleopa K.A., Sargiannidou I., Markoullis K. (2013). Connexin pathology in chronic multiple sclerosis and experimental autoimmune encephalomyelitis. Clin. Exp. Neuroimmunol..

[49] Markoullis K., Sargiannidou I., Schiza N., Hadjisavvas A., Roncaroli F., Reynolds R., Kleopa K.A. (2012). Gap junction pathology in multiple sclerosis lesions and normal-appearing white matter. Acta Neuropathol..

[50] Markoullis K., Sargiannidou I., Schiza N., Roncaroli F., Reynolds R., Kleopa K.A. (2014). Oligodendrocyte Gap Junction Loss and Disconnection from Reactive Astrocytes in Multiple Sclerosis Gray Matter. J. Neuropathol. Exp. Neurol..

[51] Markoullis K., Sargiannidou I., Gardner C., Hadjisavvas A., Reynolds R., Kleopa K.A. (2012). Disruption of oligodendrocyte gap junctions in experimental autoimmune encephalomyelitis. Glia.

[52] Papaneophytou C.P., Georgiou E., Karaiskos C., Sargiannidou I., Markoullis K., Freidin M.M., Abrams C.K., Kleopa K.A. (2018). Regulatory role of oligodendrocyte gap junctions in inflammatory demyelination. Glia.

[53] Stavropoulos F., Georgiou E., Sargiannidou I., Kleopa K.A. (2021). Dysregulation of Blood-Brain Barrier and Exacerbated Inflammatory Response in Cx47-Deficient Mice after Induction of EAE. Pharmaceuticals.

[54] Li T., Niu J., Yu G., Ezan P., Yi C., Wang X., Koulakoff A., Gao X., Chen X., Saez J.C. (2020). Connexin 43 deletion in astrocytes promotes CNS remyelination by modulating local inflammation. Glia.

[55] Zhao Y., Yamasaki R., Yamaguchi H., Nagata S., Une H., Cui Y., Masaki K., Nakamuta Y., Ionuma K., Watanabe M. (2020). Oligodendroglial connexin 47 regulates neuroinflammation upon autoimmune demyelination in a novel mouse model of multiple sclerosis. Proc. Natl. Acad. Sci. USA.

[56] Caruso G., Di Pietro L., Caraci F. (2023). Gap Junctions and Connexins in Microglia-Related Oxidative Stress and Neuroinflammation: Perspectives for Drug Discovery. Biomolecules.

[57] Juszczak G.R., Swiergiel A.H. (2009). Properties of gap junction blockers and their behavioural, cognitive and electrophysiological effects: Animal and human studies. Prog. Neuropsychopharmacol. Biol. Psychiatry.

[58] Takase E.O., Yamasaki R., Nagata S., Watanabe M., Masaki K., Yamaguchi H., Kira J.I., Takeuchi H., Isobe N. (2024). Astroglial connexin 43 is a novel therapeutic target for chronic multiple sclerosis model. Sci. Rep..

[59] Kister A., Kister I. (2023). Overview of myelin, major myelin lipids, and myelin-associated proteins. Front. Chem..

[60] Kuhn S., Gritti L., Crooks D., Dombrowski Y. (2019). Oligodendrocytes in Development, Myelin Generation and Beyond. Cells.

[61] Sun Y., Yu H., Guan Y. (2023). Glia Connect Inflammation and Neurodegeneration in Multiple Sclerosis. Neurosci. Bull..

[62] Fruhbeis C., Kuo-Elsner W.P., Muller C., Barth K., Peris L., Tenzer S., Mobius W., Werner H.B., Nave K.A., Frohlich D. (2020). Oligodendrocytes support axonal transport and maintenance via exosome secretion. PLoS Biol..

[63] Schaffner E., Bosch-Queralt M., Edgar J.M., Lehning M., Strauss J., Fleischer N., Kungl T., Wieghofer P., Berghoff S.A., Reinert T. (2023). Myelin insulation as a risk factor for axonal degeneration in autoimmune demyelinating disease. Nat. Neurosci..

[64] Dawson M.R., Polito A., Levine J.M., Reynolds R. (2003). NG2-expressing glial progenitor cells: An abundant and widespread population of cycling cells in the adult rat CNS. Mol. Cell. Neurosci..

[65] Niu J., Tsai H.H., Hoi K.K., Huang N., Yu G., Kim K., Baranzini S.E., Xiao L., Chan J.R., Fancy S.P.J. (2019). Aberrant oligodendroglial-vascular interactions disrupt the blood-brain barrier, triggering CNS inflammation. Nat. Neurosci..

[66] Tsai H.H., Niu J., Munji R., Davalos D., Chang J., Zhang H., Tien A.C., Kuo C.J., Chan J.R., Daneman R. (2016). Oligodendrocyte precursors migrate along vasculature in the developing nervous system. Science.

[67] Falcao A.M., van Bruggen D., Marques S., Meijer M., Jakel S., Agirre E., Samudyata, Floriddia E.M., Vanichkina D.P., Ffrench-Constant C. (2018). Disease-specific oligodendrocyte lineage cells arise in multiple sclerosis. Nat. Med..

[68] Jakel S., Agirre E., Mendanha Falcao A., van Bruggen D., Lee K.W., Knuesel I., Malhotra D., Ffrench-Constant C., Williams A., Castelo-Branco G. (2019). Altered human oligodendrocyte heterogeneity in multiple sclerosis. Nature.

[69] Valihrach L., Matusova Z., Zucha D., Klassen R., Benesova S., Abaffy P., Kubista M., Anderova M. (2022). Recent advances in deciphering oligodendrocyte heterogeneity with single-cell transcriptomics. Front. Cell. Neurosci..

[70] Chapman T.W., Kamen Y., Piedra E.T., Hill R.A. (2024). Oligodendrocyte Maturation Alters the Cell Death Mechanisms That Cause Demyelination. J. Neurosci. Off. J. Soc. Neurosci..

[71] Duncan I.D., Radcliff A.B., Heidari M., Kidd G., August B.K., Wierenga L.A. (2018). The adult oligodendrocyte can participate in remyelination. Proc. Natl. Acad. Sci. USA.

[72] Zeis T., Enz L., Schaeren-Wiemers N. (2016). The immunomodulatory oligodendrocyte. Brain Res..

[73] Bradl M., Lassmann H. (2010). Oligodendrocytes: Biology and pathology. Acta Neuropathol..

[74] Kirby B.B., Takada N., Latimer A.J., Shin J., Carney T.J., Kelsh R.N., Appel B. (2006). In vivo time-lapse imaging shows dynamic oligodendrocyte progenitor behavior during zebrafish development. Nat. Neurosci..

[75] Madeira M.M., Hage Z., Tsirka S.E. (2022). Beyond Myelination: Possible Roles of the Immune Proteasome in Oligodendroglial Homeostasis and Dysfunction. Front. Neurosci..

[76] Boccazzi M., Raffaele S., Fumagalli M. (2022). Not only myelination: The immuneinflammatory functions of oligodendrocytes. Neural Regen. Res..

[77] Nguyen D., Stangel M. (2001). Expression of the chemokine receptors CXCR1 and CXCR2 in rat oligodendroglial cells. Dev. Brain Res..

[78] Omari K.M., John G.R., Sealfon S.C., Raine C.S. (2005). CXC chemokine receptors on human oligodendrocytes: Implications for multiple sclerosis. Brain.

[79] Tsai H., Frost E., To V., Robinson S., Ffrench-Constant C., Geertman R., Ransohoff R.M., Miller R.H. (2002). The Chemokine Receptor CXCR2 Controls Positioning of Oligodendrocyte Precursors in Developing Spinal Cord by Arresting Their Migration. Cell.

[80] Hosking M.P., Tirotta E., Ransohoff R.M., Lane T.E. (2010). CXCR2 signaling protects oligodendrocytes and restricts demyelination in a mouse model of viral-induced demyelination. PLoS ONE.

[81] Liu L., Belkadi A., Darnall L., Hu T., Drescher C., Cotleur A.C., Padovani-Claudio D., He T., Choi K., Lane T.E. (2010). CXCR2-positive neutrophils are essential for cuprizone-induced demyelination: Relevance to multiple sclerosis. Nat. Neurosci..

[82] Patel J.R., McCandless E.E., Dorsey D., Klein R.S. (2010). CXCR4 promotes differentiation of oligodendrocyte progenitors and remyelination. Proc. Natl. Acad. Sci. USA.

[83] Tian Y., Yin H., Deng X., Tang B., Ren X., Jiang T. (2018). CXCL12 induces migration of oligodendrocyte precursor cells through the CXCR4-activated MEK/ERK and PI3K/AKT pathways. Mol. Med. Rep..

[84] Suo N., Guo Y.E., He B., Gu H., Xie X. (2019). Inhibition of MAPK/ERK pathway promotes oligodendrocytes generation and recovery of demyelinating diseases. Glia.

[85] Moyon S., Dubessy A.L., Aigrot M.S., Trotter M., Huang J.K., Dauphinot L., Potier M.C., Kerninon C., Melik Parsadaniantz S., Franklin R.J. (2015). Demyelination Causes Adult CNS Progenitors to Revert to an Immature State and Express Immune Cues That Support Their Migration. J. Neurosci..

[86] Gaffen S.L., Jain R., Garg A.V., Cua D.J. (2014). The IL-23-IL-17 immune axis: From mechanisms to therapeutic testing. Nat. Rev. Immunol..

[87] Tzartos J.S., Friese M.A., Craner M.J., Palace J., Newcombe J., Esiri M.M., Fugger L. (2008). Interleukin-17 production in central nervous system-infiltrating T cells and glial cells is associated with active disease in multiple sclerosis. Am. J. Pathol..

[88] Komiyama Y., Nakae S., Matsuki T., Nambu A., Ishigame H., Kakuta S., Sudo K., Iwakura Y. (2006). IL-17 plays an important role in the development of experimental autoimmune encephalomyelitis. J. Immunol..

[89] Zveik O., Rechtman A., Brill L., Vaknin-Dembinsky A. (2024). Anti- and pro-inflammatory milieu differentially regulate differentiation and immune functions of oligodendrocyte progenitor cells. Immunology.

[90] Kirby L., Jin J., Cardona J.G., Smith M.D., Martin K.A., Wang J., Strasburger H., Herbst L., Alexis M., Karnell J. (2019). Oligodendrocyte precursor cells present antigen and are cytotoxic targets in inflammatory demyelination. Nat. Commun..

[91] Furlan R., Brambilla E., Ruffini F., Poliani P.L., Bergami A., Marconi P.C., Franciotta D.M., Penna G., Comi G., Adorini L. (2001). Intrathecal delivery of IFN-gamma protects C57BL/6 mice from chronic-progressive experimental autoimmune encephalomyelitis by increasing apoptosis of central nervous system-infiltrating lymphocytes. J. Immunol..

[92] Gonzalez-Alvarado M.N., Aprato J., Baumeister M., Lippert M., Ekici A.B., Kirchner P., Welz T., Hoffmann A., Winkler J., Wegner M. (2022). Oligodendrocytes regulate the adhesion molecule ICAM-1 in neuroinflammation. Glia.

[93] Vautier F., Belachew S., Chittajallu R., Gallo V. (2004). Shaker-type potassium channel subunits differentially control oligodendrocyte progenitor proliferation. Glia.

[94] Gonzalez-Alvarado M.N., Rotger C., Berger L., London B., Haase S., Kuhbandner K., Lee D.H., Linker R.A. (2020). Functional role of endogenous Kv1.4 in experimental demyelination. J. Neuroimmunol..

[95] Kuhlmann T., Miron V., Cui Q., Wegner C., Antel J., Bruck W. (2008). Differentiation block of oligodendroglial progenitor cells as a cause for remyelination failure in chronic multiple sclerosis. Brain.

[96] Furusho M., Roulois A.J., Franklin R.J., Bansal R. (2015). Fibroblast growth factor signaling in oligodendrocyte-lineage cells facilitates recovery of chronically demyelinated lesions but is redundant in acute lesions. Glia.

[97] Madsen P.M., Desu H.L., de Rivero Vaccari J.P., Florimon Y., Ellman D.G., Keane R.W., Clausen B.H., Lambertsen K.L., Brambilla R. (2020). Oligodendrocytes modulate the immune-inflammatory response in EAE via TNFR2 signaling. Brain Behav. Immun..

[98] Cudrici C., Niculescu T., Niculescu F., Shin M.L., Rus H. (2006). Oligodendrocyte cell death in pathogenesis of multiple sclerosis: Protection of oligodendrocytes from apoptosis by complement. J. Rehabil. Res. Dev..

[99] Ingram G., Loveless S., Howell O.W., Hakobyan S., Dancey B., Harris C.L., Robertson N.P., Neal J.W., Morgan B.P. (2014). Complement activation in multiple sclerosis plaques: An immunohistochemical analysis. Acta Neuropathol. Commun..

[100] Liu Y., Given K.S., Harlow D.E., Matschulat A.M., Macklin W.B., Bennett J.L., Owens G.P. (2017). Myelin-specific multiple sclerosis antibodies cause complement-dependent oligodendrocyte loss and demyelination. Acta Neuropathol. Commun..

[101] Soane L., Cho H.J., Niculescu F., Rus H., Shin M.L. (2001). C5b-9 terminal complement complex protects oligodendrocytes from death by regulating Bad through phosphatidylinositol 3-kinase/Akt pathway. J. Immunol..

[102] Cudrici C., Niculescu F., Jensen T., Zafranskaia E., Fosbrink M., Rus V., Shin M.L., Rus H. (2006). C5b-9 terminal complex protects oligodendrocytes from apoptotic cell death by inhibiting caspase-8 processing and up-regulating FLIP. J. Immunol..

[103] Ghorbani S., Jelinek E., Jain R., Buehner B., Li C., Lozinski B.M., Sarkar S., Kaushik D.K., Dong Y., Wight T.N. (2022). Versican promotes T helper 17 cytotoxic inflammation and impedes oligodendrocyte precursor cell remyelination. Nat. Commun..

[104] Larochelle C., Wasser B., Jamann H., Loffel J.T., Cui Q.L., Tastet O., Schillner M., Luchtman D., Birkenstock J., Stroh A. (2021). Pro-inflammatory T helper 17 directly harms oligodendrocytes in neuroinflammation. Proc. Natl. Acad. Sci. USA.

[105] Smith K.J., Blakemore W.F., McDonald W.I. (1979). Central remyelination restores secure conduction. Nature.

[106] Mallucci G., Peruzzotti-Jametti L., Bernstock J.D., Pluchino S. (2015). The role of immune cells, glia and neurons in white and gray matter pathology in multiple sclerosis. Prog. Neurobiol..

[107] Hess K., Starost L., Kieran N.W., Thomas C., Vincenten M.C.J., Antel J., Martino G., Huitinga I., Healy L., Kuhlmann T. (2020). Lesion stage-dependent causes for impaired remyelination in MS. Acta Neuropathol..

[108] Kuhlmann T., Ludwin S., Prat A., Antel J., Bruck W., Lassmann H. (2017). An updated histological classification system for multiple sclerosis lesions. Acta Neuropathol..

[109] Moll N.M., Hong E., Fauveau M., Naruse M., Kerninon C., Tepavcevic V., Klopstein A., Seilhean D., Chew L.J., Gallo V. (2013). SOX17 is expressed in regenerating oligodendrocytes in experimental models of demyelination and in multiple sclerosis. Glia.

[110] Neely S.A., Williamson J.M., Klingseisen A., Zoupi L., Early J.J., Williams A., Lyons D.A. (2022). New oligodendrocytes exhibit more abundant and accurate myelin regeneration than those that survive demyelination. Nat. Neurosci..

[111] Macchi M., Magalon K., Zimmer C., Peeva E., El Waly B., Brousse B., Jaekel S., Grobe K., Kiefer F., Williams A. (2020). Mature oligodendrocytes bordering lesions limit demyelination and favor myelin repair via heparan sulfate production. eLife.

[112] Zawadzka M., Rivers L.E., Fancy S.P., Zhao C., Tripathi R., Jamen F., Young K., Goncharevich A., Pohl H., Rizzi M. (2010). CNS-resident glial progenitor/stem cells produce Schwann cells as well as oligodendrocytes during repair of CNS demyelination. Cell Stem Cell.

[113] Kirby L., Castelo-Branco G. (2021). Crossing boundaries: Interplay between the immune system and oligodendrocyte lineage cells. Semin. Cell Dev. Biol..

[114] Franklin R.J.M., Simons M. (2022). CNS remyelination and inflammation: From basic mechanisms to therapeutic opportunities. Neuron.

[115] Marangon D., Castro E.S.J.H., Cerrato V., Boda E., Lecca D. (2024). Oligodendrocyte Progenitors in Glial Scar: A Bet on Remyelination. Cells.

[116] Singh J., Sharma K., Frost E.E., Pillai P.P. (2019). Role of PDGF-A-Activated ERK Signaling Mediated FAK-Paxillin Interaction in Oligodendrocyte Progenitor Cell Migration. J. Mol. Neurosci..

[117] Ghorbani S., Yong V.W. (2021). The extracellular matrix as modifier of neuroinflammation and remyelination in multiple sclerosis. Brain.

[118] Fancy S.P., Baranzini S.E., Zhao C., Yuk D.I., Irvine K.A., Kaing S., Sanai N., Franklin R.J., Rowitch D.H. (2009). Dysregulation of the Wnt pathway inhibits timely myelination and remyelination in the mammalian CNS. Genes. Dev..

[119] Wang S., Sdrulla A.D., diSibio G., Bush G., Nofziger D., Hicks C., Weinmaster G., Barres B.A. (1998). Notch receptor activation inhibits oligodendrocyte differentiation. Neuron.

[120] Wang J., Saraswat D., Sinha A.K., Polanco J., Dietz K., O’Bara M.A., Pol S.U., Shayya H.J., Sim F.J. (2018). Paired Related Homeobox Protein 1 Regulates Quiescence in Human Oligodendrocyte Progenitors. Cell Rep..

[121] Tepavcevic V., Blakemore W.F. (2005). Glial grafting for demyelinating disease. Philos. Trans. R. Soc. Lond. B Biol. Sci..

[122] Lin K., Bieri G., Gontier G., Muller S., Smith L.K., Snethlage C.E., White C.W., Maybury-Lewis S.Y., Villeda S.A. (2021). MHC class I H2-Kb negatively regulates neural progenitor cell proliferation by inhibiting FGFR signaling. PLoS Biol..

[123] Kaya T., Mattugini N., Liu L., Ji H., Cantuti-Castelvetri L., Wu J., Schifferer M., Groh J., Martini R., Besson-Girard S. (2022). CD8^+^ T cells induce interferon-responsive oligodendrocytes and microglia in white matter aging. Nat. Neurosci..

[124] Tepavcevic V., Lubetzki C. (2022). Oligodendrocyte progenitor cell recruitment and remyelination in multiple sclerosis: The more, the merrier?. Brain.

[125] Baxi E.G., DeBruin J., Tosi D.M., Grishkan I.V., Smith M.D., Kirby L.A., Strasburger H.J., Fairchild A.N., Calabresi P.A., Gocke A.R. (2015). Transfer of myelin-reactive th17 cells impairs endogenous remyelination in the central nervous system of cuprizone-fed mice. J. Neurosci. Off. J. Soc. Neurosci..

[126] Choi E.H., Xu Y., Medynets M., Monaco M.C.G., Major E.O., Nath A., Wang T. (2018). Activated T cells induce proliferation of oligodendrocyte progenitor cells via release of vascular endothelial cell growth factor-A. Glia.

[127] Dombrowski Y., O’Hagan T., Dittmer M., Penalva R., Mayoral S.R., Bankhead P., Fleville S., Eleftheriadis G., Zhao C., Naughton M. (2017). Regulatory T cells promote myelin regeneration in the central nervous system. Nat. Neurosci..

[128] Mei F., Fancy S.P.J., Shen Y.A., Niu J., Zhao C., Presley B., Miao E., Lee S., Mayoral S.R., Redmond S.A. (2014). Micropillar arrays as a high-throughput screening platform for therapeutics in multiple sclerosis. Nat. Med..

[129] Deshmukh V.A., Tardif V., Lyssiotis C.A., Green C.C., Kerman B., Kim H.J., Padmanabhan K., Swoboda J.G., Ahmad I., Kondo T. (2013). A regenerative approach to the treatment of multiple sclerosis. Nature.

[130] Li W., Berlinicke C., Huang Y., Giera S., McGrath A.G., Fang W., Chen C., Takaesu F., Chang X., Duan Y. (2023). High-throughput screening for myelination promoting compounds using human stem cell-derived oligodendrocyte progenitor cells. iScience.

[131] Iliff J.J., Wang M., Liao Y., Plogg B.A., Peng W., Gundersen G.A., Benveniste H., Vates G.E., Deane R., Goldman S.A. (2012). A paravascular pathway facilitates CSF flow through the brain parenchyma and the clearance of interstitial solutes, including amyloid beta. Sci. Transl. Med..

[132] Mader S., Brimberg L. (2019). Aquaporin-4 Water Channel in the Brain and Its Implication for Health and Disease. Cells.

[133] Diaz-Castro B., Robel S., Mishra A. (2023). Astrocyte Endfeet in Brain Function and Pathology: Open Questions. Annu. Rev. Neurosci..

[134] Stevens B., Allen N.J., Vazquez L.E., Howell G.R., Christopherson K.S., Nouri N., Micheva K.D., Mehalow A.K., Huberman A.D., Stafford B. (2007). The classical complement cascade mediates CNS synapse elimination. Cell.

[135] Morizawa Y.M., Hirayama Y., Ohno N., Shibata S., Shigetomi E., Sui Y., Nabekura J., Sato K., Okajima F., Takebayashi H. (2017). Reactive astrocytes function as phagocytes after brain ischemia via ABCA1-mediated pathway. Nat. Commun..

[136] Patani R., Hardingham G.E., Liddelow S.A. (2023). Functional roles of reactive astrocytes in neuroinflammation and neurodegeneration. Nat. Rev. Neurol..

[137] Molofsky A.V., Deneen B. (2015). Astrocyte development: A Guide for the Perplexed. Glia.

[138] Cajal S.R.Y., Azoulay L. (1909). Histologie du Système Nerveux de L’homme et des Vertébrés.

[139] Absinta M., Maric D., Gharagozloo M., Garton T., Smith M.D., Jin J., Fitzgerald K.C., Song A., Liu P., Lin J.P. (2021). A lymphocyte-microglia-astrocyte axis in chronic active multiple sclerosis. Nature.

[140] Liddelow S.A., Guttenplan K.A., Clarke L.E., Bennett F.C., Bohlen C.J., Schirmer L., Bennett M.L., Munch A.E., Chung W.S., Peterson T.C. (2017). Neurotoxic reactive astrocytes are induced by activated microglia. Nature.

[141] Das Neves S.P., Sousa J.C., Magalhaes R., Gao F., Coppola G., Meriaux S., Boumezbeur F., Sousa N., Cerqueira J.J., Marques F. (2023). Astrocytes Undergo Metabolic Reprogramming in the Multiple Sclerosis Animal Model. Cells.

[142] Brambilla R., Bracchi-Ricard V., Hu W.H., Frydel B., Bramwell A., Karmally S., Green E.J., Bethea J.R. (2005). Inhibition of astroglial nuclear factor kappaB reduces inflammation and improves functional recovery after spinal cord injury. J. Exp. Med..

[143] Ceyzeriat K., Abjean L., Carrillo-de Sauvage M.A., Ben Haim L., Escartin C. (2016). The complex STATes of astrocyte reactivity: How are they controlled by the JAK-STAT3 pathway?. Neuroscience.

[144] Choudhury R.G., Ryou M.G., Poteet E., Wen Y., He R., Sun F., Yuan F., Jin K., Yang S.H. (2014). Involvement of p38 MAPK in reactive astrogliosis induced by ischemic stroke. Brain Res..

[145] Blank T., Prinz M. (2014). NF-kappaB signaling regulates myelination in the CNS. Front. Mol. Neurosci..

[146] Brambilla R., Hurtado A., Persaud T., Esham K., Pearse D.D., Oudega M., Bethea J.R. (2009). Transgenic inhibition of astroglial NF-kappa B leads to increased axonal sparing and sprouting following spinal cord injury. J. Neurochem..

[147] Kang Z., Liu L., Spangler R., Spear C., Wang C., Gulen M.F., Veenstra M., Ouyang W., Ransohoff R.M., Li X. (2012). IL-17-induced Act1-mediated signaling is critical for cuprizone-induced demyelination. J. Neurosci. Off. J. Soc. Neurosci..

[148] Kang Z., Wang C., Zepp J., Wu L., Sun K., Zhao J., Chandrasekharan U., DiCorleto P.E., Trapp B.D., Ransohoff R.M. (2013). Act1 mediates IL-17-induced EAE pathogenesis selectively in NG2+ glial cells. Nat. Neurosci..

[149] Guerrero-García J.J. (2020). The role of astrocytes in multiple sclerosis pathogenesis. Neurol. Engl. Ed..

[150] Boccazzi M., Van Steenwinckel J., Schang A.L., Faivre V., Le Charpentier T., Bokobza C., Csaba Z., Verderio C., Fumagalli M., Mani S. (2021). The immune-inflammatory response of oligodendrocytes in a murine model of preterm white matter injury: The role of TLR3 activation. Cell Death Dis..

[151] Gambuzza M., Licata N., Palella E., Celi D., Foti Cuzzola V., Italiano D., Marino S., Bramanti P. (2011). Targeting Toll-like receptors: Emerging therapeutics for multiple sclerosis management. J. Neuroimmunol..

[152] Bsibsi M., Persoon-Deen C., Verwer R.W., Meeuwsen S., Ravid R., Van Noort J.M. (2006). Toll-like receptor 3 on adult human astrocytes triggers production of neuroprotective mediators. Glia.

[153] Itoh N., Itoh Y., Tassoni A., Ren E., Kaito M., Ohno A., Ao Y., Farkhondeh V., Johnsonbaugh H., Burda J. (2018). Cell-specific and region-specific transcriptomics in the multiple sclerosis model: Focus on astrocytes. Proc. Natl. Acad. Sci. USA.

[154] Kramann N., Menken L., Pfortner R., Schmid S.N., Stadelmann C., Wegner C., Bruck W. (2019). Glial fibrillary acidic protein expression alters astrocytic chemokine release and protects mice from cuprizone-induced demyelination. Glia.

[155] Schiera G., Di Liegro C.M., Schiro G., Sorbello G., Di Liegro I. (2024). Involvement of Astrocytes in the Formation, Maintenance, and Function of the Blood-Brain Barrier. Cells.

[156] Sharma R., Fischer M.T., Bauer J., Felts P.A., Smith K.J., Misu T., Fujihara K., Bradl M., Lassmann H. (2010). Inflammation induced by innate immunity in the central nervous system leads to primary astrocyte dysfunction followed by demyelination. Acta Neuropathol..

[157] Parratt J.D., Prineas J.W. (2010). Neuromyelitis optica: A demyelinating disease characterized by acute destruction and regeneration of perivascular astrocytes. Mult. Scler..

[158] Gimenez M.A., Sim J.E., Russell J.H. (2004). TNFR1-dependent VCAM-1 expression by astrocytes exposes the CNS to destructive inflammation. J. Neuroimmunol..

[159] Popescu B., Guo Y., Jentoft M.E., Parisi J.E., Lennon V.A., Pittock S.J., Weinshenker B.G., Wingerchuk D.M., Giannini C., Metz I. (2015). Diagnostic utility of aquaporin-4 in the analysis of active demyelinating lesions. Neurology.

[160] Miyamoto K., Nagaosa N., Motoyama M., Kataoka K., Kusunoki S. (2009). Upregulation of water channel aquaporin-4 in experimental autoimmune encephalomyeritis. J. Neurol. Sci..

[161] Li L., Zhang H., Verkman A.S. (2009). Greatly attenuated experimental autoimmune encephalomyelitis in aquaporin-4 knockout mice. BMC Neurosci..

[162] Kim R.Y., Hoffman A.S., Itoh N., Ao Y., Spence R., Sofroniew M.V., Voskuhl R.R. (2014). Astrocyte CCL2 sustains immune cell infiltration in chronic experimental autoimmune encephalomyelitis. J. Neuroimmunol..

[163] Kunkl M., Amormino C., Tedeschi V., Fiorillo M.T., Tuosto L. (2022). Astrocytes and Inflammatory T Helper Cells: A Dangerous Liaison in Multiple Sclerosis. Front. Immunol..

[164] Correale J., Farez M.F. (2015). The Role of Astrocytes in Multiple Sclerosis Progression. Front. Neurol..

[165] Damsker J.M., Hansen A.M., Caspi R.R. (2010). Th1 and Th17 cells: Adversaries and collaborators. Ann. N. Y. Acad. Sci..

[166] Prajeeth C.K., Kronisch J., Khorooshi R., Knier B., Toft-Hansen H., Gudi V., Floess S., Huehn J., Owens T., Korn T. (2017). Effectors of Th1 and Th17 cells act on astrocytes and augment their neuroinflammatory properties. J. Neuroinflammation.

[167] McWilliams I.L., Rajbhandari R., Nozell S., Benveniste E., Harrington L.E. (2015). STAT4 controls GM-CSF production by both Th1 and Th17 cells during EAE. J. Neuroinflammation.

[168] Kostic M., Zivkovic N., Cvetanovic A., Stojanovic I. (2018). Granulocyte-macrophage colony-stimulating factor as a mediator of autoimmunity in multiple sclerosis. J. Neuroimmunol..

[169] Watanabe M., Masaki K., Yamasaki R., Kawanokuchi J., Takeuchi H., Matsushita T., Suzumura A., Kira J.I. (2016). Th1 cells downregulate connexin 43 gap junctions in astrocytes via microglial activation. Sci. Rep..

[170] Senecal V., Deblois G., Beauseigle D., Schneider R., Brandenburg J., Newcombe J., Moore C.S., Prat A., Antel J., Arbour N. (2016). Production of IL-27 in multiple sclerosis lesions by astrocytes and myeloid cells: Modulation of local immune responses. Glia.

[171] Lemaitre F., Farzam-Kia N., Carmena Moratalla A., Carpentier Solorio Y., Clenet M.L., Tastet O., Cleret-Buhot A., Guimond J.V., Haddad E., Duquette P. (2022). IL-27 shapes the immune properties of human astrocytes and their impact on encountered human T lymphocytes. J. Neuroinflammation.

[172] Clarner T., Janssen K., Nellessen L., Stangel M., Skripuletz T., Krauspe B., Hess F.M., Denecke B., Beutner C., Linnartz-Gerlach B. (2015). CXCL10 triggers early microglial activation in the cuprizone model. J. Immunol..

[173] Mayo L., Trauger S.A., Blain M., Nadeau M., Patel B., Alvarez J.I., Mascanfroni I.D., Yeste A., Kivisäkk P., Kallas K. (2014). Regulation of astrocyte activation by glycolipids drives chronic CNS inflammation. Nat. Med..

[174] Tenner A.J., Stevens B., Woodruff T.M. (2018). New tricks for an ancient system: Physiological and pathological roles of complement in the CNS. Mol. Immunol..

[175] Gasque P., Singhrao S.K., Neal J.W., Gotze O., Morgan B.P. (1997). Expression of the receptor for complement C5a (CD88) is up-regulated on reactive astrocytes, microglia, and endothelial cells in the inflamed human central nervous system. Am. J. Pathol..

[176] Crane J.W., Baiquni G.P., Sullivan R.K., Lee J.D., Sah P., Taylor S.M., Noakes P.G., Woodruff T.M. (2009). The C5a anaphylatoxin receptor CD88 is expressed in presynaptic terminals of hippocampal mossy fibres. J. Neuroinflammation.

[177] Loveless S., Neal J.W., Howell O.W., Harding K.E., Sarkies P., Evans R., Bevan R.J., Hakobyan S., Harris C.L., Robertson N.P. (2018). Tissue microarray methodology identifies complement pathway activation and dysregulation in progressive multiple sclerosis. Brain Pathol..

[178] Olivero G., Taddeucci A., Vallarino G., Trebesova H., Roggeri A., Gagliani M.C., Cortese K., Grilli M., Pittaluga A. (2024). Complement tunes glutamate release and supports synaptic impairments in an animal model of multiple sclerosis. Br. J. Pharmacol..

[179] Schroder L.J., Mulenge F., Pavlou A., Skripuletz T., Stangel M., Gudi V., Kalinke U. (2023). Dynamics of reactive astrocytes fosters tissue regeneration after cuprizone-induced demyelination. Glia.

[180] Rawji K.S., Gonzalez Martinez G.A., Sharma A., Franklin R.J.M. (2020). The Role of Astrocytes in Remyelination. Trends Neurosci..

[181] Hammond T.R., Gadea A., Dupree J., Kerninon C., Nait-Oumesmar B., Aguirre A., Gallo V. (2014). Astrocyte-derived endothelin-1 inhibits remyelination through notch activation. Neuron.

[182] Hammond T.R., McEllin B., Morton P.D., Raymond M., Dupree J., Gallo V. (2015). Endothelin-B Receptor Activation in Astrocytes Regulates the Rate of Oligodendrocyte Regeneration during Remyelination. Cell Rep..

[183] Watzlawik J.O., Warrington A.E., Rodriguez M. (2013). PDGF is required for remyelination-promoting IgM stimulation of oligodendrocyte progenitor cell proliferation. PLoS ONE.

[184] Thummler K., Rom E., Zeis T., Lindner M., Brunner S., Cole J.J., Arseni D., Mucklisch S., Edgar J.M., Schaeren-Wiemers N. (2019). Polarizing receptor activation dissociates fibroblast growth factor 2 mediated inhibition of myelination from its neuroprotective potential. Acta Neuropathol. Commun..

[185] Skripuletz T., Hackstette D., Bauer K., Gudi V., Pul R., Voss E., Berger K., Kipp M., Baumgartner W., Stangel M. (2013). Astrocytes regulate myelin clearance through recruitment of microglia during cuprizone-induced demyelination. Brain.

[186] Moore C.S., Abdullah S.L., Brown A., Arulpragasam A., Crocker S.J. (2011). How factors secreted from astrocytes impact myelin repair. J. Neurosci. Res..

[187] Cheng N., Xiong Y., Zhang W., Wu X., Sun Z., Zhang L., Wu H., Tang Y., Peng Y. (2022). Astrocytes promote the proliferation of oligodendrocyte precursor cells through connexin 47-mediated LAMB2 secretion in exosomes. Mol. Biol. Rep..

[188] Xu D., Liu Z., Wang S., Peng Y., Sun X. (2017). Astrocytes regulate the expression of Sp1R3 on oligodendrocyte progenitor cells through Cx47 and promote their proliferation. Biochem. Biophys. Res. Commun..

[189] Silva Oliveira Junior M., Reiche L., Daniele E., Kortebi I., Faiz M., Kury P. (2024). Star power: Harnessing the reactive astrocyte response to promote remyelination in multiple sclerosis. Neural Regen. Res..

[190] Yang S., Qin C., Hu Z.W., Zhou L.Q., Yu H.H., Chen M., Bosco D.B., Wang W., Wu L.J., Tian D.S. (2021). Microglia reprogram metabolic profiles for phenotype and function changes in central nervous system. Neurobiol. Dis..

[191] McNamara N.B., Munro D.A.D., Bestard-Cuche N., Uyeda A., Bogie J.F.J., Hoffmann A., Holloway R.K., Molina-Gonzalez I., Askew K.E., Mitchell S. (2023). Microglia regulate central nervous system myelin growth and integrity. Nature.

[192] Olcum M., Tastan B., Kiser C., Genc S., Genc K. (2020). Microglial NLRP3 inflammasome activation in multiple sclerosis. Adv. Protein Chem. Struct. Biol..

[193] Andoh M., Koyama R. (2021). Microglia regulate synaptic development and plasticity. Dev. Neurobiol..

[194] Cornell J., Salinas S., Huang H.Y., Zhou M. (2022). Microglia regulation of synaptic plasticity and learning and memory. Neural Regen. Res..

[195] Zhou L.J., Peng J., Xu Y.N., Zeng W.J., Zhang J., Wei X., Mai C.L., Lin Z.J., Liu Y., Murugan M. (2019). Microglia Are Indispensable for Synaptic Plasticity in the Spinal Dorsal Horn and Chronic Pain. Cell Rep..

[196] Colonna M., Butovsky O. (2017). Microglia Function in the Central Nervous System During Health and Neurodegeneration. Annu. Rev. Immunol..

[197] Savage J.C., Carrier M., Tremblay M.E. (2019). Morphology of Microglia Across Contexts of Health and Disease. Methods Mol. Biol..

[198] Streit W.J., Mrak R.E., Griffin W.S. (2004). Microglia and neuroinflammation: A pathological perspective. J. Neuroinflammation.

[199] Adamu A., Li S., Gao F., Xue G. (2024). The role of neuroinflammation in neurodegenerative diseases: Current understanding and future therapeutic targets. Front. Aging Neurosci..

[200] Lavin Y., Winter D., Blecher-Gonen R., David E., Keren-Shaul H., Merad M., Jung S., Amit I. (2014). Tissue-resident macrophage enhancer landscapes are shaped by the local microenvironment. Cell.

[201] Zhang X., Chen F., Sun M., Wu N., Liu B., Yi X., Ge R., Fan X. (2023). Microglia in the context of multiple sclerosis. Front. Neurol..

[202] Hou K., Li G., Yu J., Xu K., Wu W. (2021). Receptors, Channel Proteins, and Enzymes Involved in Microglia-mediated Neuroinflammation and Treatments by Targeting Microglia in Ischemic Stroke. Neuroscience.

[203] Guo S., Wang H., Yin Y. (2022). Microglia Polarization from M1 to M2 in Neurodegenerative Diseases. Front. Aging Neurosci..

[204] Bottcher J.P., Bonavita E., Chakravarty P., Blees H., Cabeza-Cabrerizo M., Sammicheli S., Rogers N.C., Sahai E., Zelenay S., Reis e Sousa C. (2018). NK Cells Stimulate Recruitment of cDC1 into the Tumor Microenvironment Promoting Cancer Immune Control. Cell.

[205] Zia S., Rawji K.S., Michaels N.J., Burr M., Kerr B.J., Healy L.M., Plemel J.R. (2020). Microglia Diversity in Health and Multiple Sclerosis. Front. Immunol..

[206] Van den Bosch A.M.R., van der Poel M., Fransen N.L., Vincenten M.C.J., Bobeldijk A.M., Jongejan A., Engelenburg H.J., Moerland P.D., Smolders J., Huitinga I. (2024). Profiling of microglia nodules in multiple sclerosis reveals propensity for lesion formation. Nat. Commun..

[207] Marzan D.E., Brugger-Verdon V., West B.L., Liddelow S., Samanta J., Salzer J.L. (2021). Activated microglia drive demyelination via CSF1R signaling. Glia.

[208] Peruzzotti-Jametti L., Willis C.M., Krzak G., Hamel R., Pirvan L., Ionescu R.B., Reisz J.A., Prag H.A., Garcia-Segura M.E., Wu V. (2024). Mitochondrial complex I activity in microglia sustains neuroinflammation. Nature.

[209] Zrzavy T., Hametner S., Wimmer I., Butovsky O., Weiner H.L., Lassmann H. (2017). Loss of ‘homeostatic’ microglia and patterns of their activation in active multiple sclerosis. Brain.

[210] Vogel D.Y., Vereyken E.J., Glim J.E., Heijnen P.D., Moeton M., van der Valk P., Amor S., Teunissen C.E., van Horssen J., Dijkstra C.D. (2013). Macrophages in inflammatory multiple sclerosis lesions have an intermediate activation status. J. Neuroinflammation.

[211] O’Loughlin E., Madore C., Lassmann H., Butovsky O. (2018). Microglial Phenotypes and Functions in Multiple Sclerosis. Cold Spring Harb. Perspect. Med..

[212] Nowacki P., Koziarska D., Masztalewicz M. (2019). Microglia and astroglia proliferation within the normal appearing white matter in histologically active and inactive multiple sclerosis. Folia Neuropathol..

[213] Guerrero B.L., Sicotte N.L. (2020). Microglia in Multiple Sclerosis: Friend or Foe?. Front. Immunol..

[214] Mado H., Adamczyk-Sowa M., Sowa P. (2023). Role of Microglial Cells in the Pathophysiology MS: Synergistic or Antagonistic?. Int. J. Mol. Sci..

[215] Seo J.E., Hasan M., Han J.S., Kang M.J., Jung B.H., Kwok S.K., Kim H.Y., Kwon O.S. (2015). Experimental autoimmune encephalomyelitis and age-related correlations of NADPH oxidase, MMP-9, and cell adhesion molecules: The increased disease severity and blood-brain barrier permeability in middle-aged mice. J. Neuroimmunol..

[216] Subbarayan M.S., Joly-Amado A., Bickford P.C., Nash K.R. (2022). CX3CL1/CX3CR1 signaling targets for the treatment of neurodegenerative diseases. Pharmacol. Ther..

[217] Lampron A., Larochelle A., Laflamme N., Prefontaine P., Plante M.M., Sanchez M.G., Yong V.W., Stys P.K., Tremblay M.E., Rivest S. (2015). Inefficient clearance of myelin debris by microglia impairs remyelinating processes. J. Exp. Med..

[218] Wasser B., Luchtman D., Loffel J., Robohm K., Birkner K., Stroh A., Vogelaar C.F., Zipp F., Bittner S. (2020). CNS-localized myeloid cells capture living invading T cells during neuroinflammation. J. Exp. Med..

[219] Moser T., Akgun K., Proschmann U., Sellner J., Ziemssen T. (2020). The role of TH17 cells in multiple sclerosis: Therapeutic implications. Autoimmun. Rev..

[220] Van Langelaar J., van der Vuurst de Vries R.M., Janssen M., Wierenga-Wolf A.F., Spilt I.M., Siepman T.A., Dankers W., Verjans G., de Vries H.E., Lubberts E. (2018). T helper 17.1 cells associate with multiple sclerosis disease activity: Perspectives for early intervention. Brain.

[221] Strachan-Whaley M., Rivest S., Yong V.W. (2014). Interactions between microglia and T cells in multiple sclerosis pathobiology. J. Interferon Cytokine Res..

[222] Dong Y., Yong V.W. (2019). When encephalitogenic T cells collaborate with microglia in multiple sclerosis. Nat. Rev. Neurol..

[223] Kalafatakis I., Karagogeos D. (2021). Oligodendrocytes and Microglia: Key Players in Myelin Development, Damage and Repair. Biomolecules.

[224] Prajeeth C.K., Lohr K., Floess S., Zimmermann J., Ulrich R., Gudi V., Beineke A., Baumgartner W., Muller M., Huehn J. (2014). Effector molecules released by Th1 but not Th17 cells drive an M1 response in microglia. Brain Behav. Immun..

[225] Kunkl M., Frascolla S., Amormino C., Volpe E., Tuosto L. (2020). T Helper Cells: The Modulators of Inflammation in Multiple Sclerosis. Cells.

[226] Sloane J.A., Batt C., Ma Y., Harris Z.M., Trapp B., Vartanian T. (2010). Hyaluronan blocks oligodendrocyte progenitor maturation and remyelination through TLR2. Proc. Natl. Acad. Sci. USA.

[227] Andersson J., Tran D.Q., Pesu M., Davidson T.S., Ramsey H., O’Shea J.J., Shevach E.M. (2008). CD4+ FoxP3+ regulatory T cells confer infectious tolerance in a TGF-beta-dependent manner. J. Exp. Med..

[228] Calvo-Rodriguez M., Garcia-Rodriguez C., Villalobos C., Nunez L. (2020). Role of Toll Like Receptor 4 in Alzheimer’s Disease. Front. Immunol..

[229] Miranda-Hernandez S., Baxter A.G. (2013). Role of toll-like receptors in multiple sclerosis. Am. J. Clin. Exp. Immunol..

[230] Fornari Laurindo L., Aparecido Dias J., Cressoni Araujo A., Torres Pomini K., Machado Galhardi C., Rucco Penteado Detregiachi C., Santos de Argollo Haber L., Donizeti Roque D., Dib Bechara M., Vialogo Marques de Castro M. (2023). Immunological dimensions of neuroinflammation and microglial activation: Exploring innovative immunomodulatory approaches to mitigate neuroinflammatory progression. Front. Immunol..

[231] Zhong Z., Umemura A., Sanchez-Lopez E., Liang S., Shalapour S., Wong J., He F., Boassa D., Perkins G., Ali S.R. (2016). NF-kappaB Restricts Inflammasome Activation via Elimination of Damaged Mitochondria. Cell.

[232] Lee M.J., Bing S.J., Choi J., Jang M., Lee G., Lee H., Chang B.S., Jee Y., Lee S.J., Cho I.H. (2016). IKKbeta-mediated inflammatory myeloid cell activation exacerbates experimental autoimmune encephalomyelitis by potentiating Th1/Th17 cell activation and compromising blood brain barrier. Mol. Neurodegener..

[233] Krasemann S., Madore C., Cialic R., Baufeld C., Calcagno N., El Fatimy R., Beckers L., O’Loughlin E., Xu Y., Fanek Z. (2017). The TREM2-APOE Pathway Drives the Transcriptional Phenotype of Dysfunctional Microglia in Neurodegenerative Diseases. Immunity.

[234] Hagan N., Kane J.L., Grover D., Woodworth L., Madore C., Saleh J., Sancho J., Liu J., Li Y., Proto J. (2020). CSF1R signaling is a regulator of pathogenesis in progressive MS. Cell Death Dis..

[235] Bottcher C., Fernandez-Zapata C., Schlickeiser S., Kunkel D., Schulz A.R., Mei H.E., Weidinger C., Giess R.M., Asseyer S., Siegmund B. (2019). Multi-parameter immune profiling of peripheral blood mononuclear cells by multiplexed single-cell mass cytometry in patients with early multiple sclerosis. Sci. Rep..

[236] Aloisi F., Serafini B., Adorini L. (2000). Glia-T cell dialogue. J. Neuroimmunol..

[237] Fresegna D., Bullitta S., Musella A., Rizzo F.R., De Vito F., Guadalupi L., Caioli S., Balletta S., Sanna K., Dolcetti E. (2020). Re-Examining the Role of TNF in MS Pathogenesis and Therapy. Cells.

[238] Raffaele S., Lombardi M., Verderio C., Fumagalli M. (2020). TNF Production and Release from Microglia via Extracellular Vesicles: Impact on Brain Functions. Cells.

[239] Guadalupi L., Vanni V., Balletta S., Caioli S., De Vito F., Fresegna D., Sanna K., Nencini M., Donninelli G., Volpe E. (2024). Interleukin-9 protects from microglia- and TNF-mediated synaptotoxicity in experimental multiple sclerosis. J. Neuroinflammation.

[240] Batoulis H., Recks M.S., Holland F.O., Thomalla F., Williams R.O., Kuerten S. (2014). Blockade of tumour necrosis factor-alpha in experimental autoimmune encephalomyelitis reveals differential effects on the antigen-specific immune response and central nervous system histopathology. Clin. Exp. Immunol..

[241] Deczkowska A., Baruch K., Schwartz M. (2016). Type I/II Interferon Balance in the Regulation of Brain Physiology and Pathology. Trends Immunol..

[242] Takeuchi H., Wang J., Kawanokuchi J., Mitsuma N., Mizuno T., Suzumura A. (2006). Interferon-gamma induces microglial-activation-induced cell death: A hypothetical mechanism of relapse and remission in multiple sclerosis. Neurobiol. Dis..

[243] Papageorgiou I.E., Lewen A., Galow L.V., Cesetti T., Scheffel J., Regen T., Hanisch U.K., Kann O. (2016). TLR4-activated microglia require IFN-gamma to induce severe neuronal dysfunction and death in situ. Proc. Natl. Acad. Sci. USA.

[244] Tichauer J.E., Arellano G., Acuna E., Gonzalez L.F., Kannaiyan N.R., Murgas P., Panadero-Medianero C., Ibanez-Vega J., Burgos P.I., Loda E. (2023). Interferon-gamma ameliorates experimental autoimmune encephalomyelitis by inducing homeostatic adaptation of microglia. Front. Immunol..

[245] Wies Mancini V.S.B., Mattera V.S., Pasquini J.M., Pasquini L.A., Correale J.D. (2024). Microglia-derived extracellular vesicles in homeostasis and demyelination/remyelination processes. J. Neurochem..

[246] Mahmood A., Miron V.E. (2022). Microglia as therapeutic targets for central nervous system remyelination. Curr. Opin. Pharmacol..

[247] Hammond T.R., Dufort C., Dissing-Olesen L., Giera S., Young A., Wysoker A., Walker A.J., Gergits F., Segel M., Nemesh J. (2019). Single-Cell RNA Sequencing of Microglia throughout the Mouse Lifespan and in the Injured Brain Reveals Complex Cell-State Changes. Immunity.

[248] Miron V.E., Boyd A., Zhao J.W., Yuen T.J., Ruckh J.M., Shadrach J.L., van Wijngaarden P., Wagers A.J., Williams A., Franklin R.J.M. (2013). M2 microglia and macrophages drive oligodendrocyte differentiation during CNS remyelination. Nat. Neurosci..

[249] Zarini D., Pasbakhsh P., Mojaverrostami S., Amirizadeh S., Hashemi M., Shabani M., Noshadian M., Kashani I.R. (2024). Microglia/macrophage polarization regulates spontaneous remyelination in intermittent cuprizone model of demyelination. Biochem. Biophys. Rep..

[250] Lloyd A.F., Miron V.E. (2019). The pro-remyelination properties of microglia in the central nervous system. Nat. Rev. Neurol..

[251] Lloyd A.F., Davies C.L., Holloway R.K., Labrak Y., Ireland G., Carradori D., Dillenburg A., Borger E., Soong D., Richardson J.C. (2019). Central nervous system regeneration is driven by microglia necroptosis and repopulation. Nat. Neurosci..

[252] Plemel J.R., Manesh S.B., Sparling J.S., Tetzlaff W. (2013). Myelin inhibits oligodendroglial maturation and regulates oligodendrocytic transcription factor expression. Glia.

[253] Kotter M.R., Setzu A., Sim F.J., Van Rooijen N., Franklin R.J. (2001). Macrophage depletion impairs oligodendrocyte remyelination following lysolecithin-induced demyelination. Glia.

[254] Kotter M.R., Zhao C., van Rooijen N., Franklin R.J. (2005). Macrophage-depletion induced impairment of experimental CNS remyelination is associated with a reduced oligodendrocyte progenitor cell response and altered growth factor expression. Neurobiol. Dis..

[255] Ruckh J.M., Zhao J.W., Shadrach J.L., van Wijngaarden P., Rao T.N., Wagers A.J., Franklin R.J. (2012). Rejuvenation of regeneration in the aging central nervous system. Cell Stem Cell.

[256] Berghoff S.A., Spieth L., Sun T., Hosang L., Schlaphoff L., Depp C., Duking T., Winchenbach J., Neuber J., Ewers D. (2021). Microglia facilitate repair of demyelinated lesions via post-squalene sterol synthesis. Nat. Neurosci..

[257] Laflamme N., Cisbani G., Prefontaine P., Srour Y., Bernier J., St-Pierre M.K., Tremblay M.E., Rivest S. (2018). mCSF-Induced Microglial Activation Prevents Myelin Loss and Promotes Its Repair in a Mouse Model of Multiple Sclerosis. Front. Cell. Neurosci..

[258] Church J.S., Kigerl K.A., Lerch J.K., Popovich P.G., McTigue D.M. (2016). TLR4 Deficiency Impairs Oligodendrocyte Formation in the Injured Spinal Cord. J. Neurosci. Off. J. Soc. Neurosci..

[259] Wasko N.J., Kulak M.H., Paul D., Nicaise A.M., Yeung S.T., Nichols F.C., Khanna K.M., Crocker S., Pachter J.S., Clark R.B. (2019). Systemic TLR2 tolerance enhances central nervous system remyelination. J. Neuroinflammation.

[260] Miron V.E. (2017). Microglia-driven regulation of oligodendrocyte lineage cells, myelination, and remyelination. J. Leukoc. Biol..

[261] Williams A., Piaton G., Aigrot M.S., Belhadi A., Theaudin M., Petermann F., Thomas J.L., Zalc B., Lubetzki C. (2007). Semaphorin 3A and 3F: Key players in myelin repair in multiple sclerosis?. Brain.

[262] Arnett H.A., Mason J., Marino M., Suzuki K., Matsushima G.K., Ting J.P. (2001). TNF alpha promotes proliferation of oligodendrocyte progenitors and remyelination. Nat. Neurosci..

[263] Hlavica M., Delparente A., Good A., Good N., Plattner P.S., Seyedsadr M.S., Schwab M.E., Figlewicz D.P., Ineichen B.V. (2017). Intrathecal insulin-like growth factor 1 but not insulin enhances myelin repair in young and aged rats. Neurosci. Lett..

[264] Mason J.L., Suzuki K., Chaplin D.D., Matsushima G.K. (2001). Interleukin-1beta promotes repair of the CNS. J. Neurosci. Off. J. Soc. Neurosci..

[265] Baaklini C.S., Ho M.F.S., Lange T., Hammond B.P., Panda S.P., Zirngibl M., Zia S., Himmelsbach K., Rana H., Phillips B. (2023). Microglia promote remyelination independent of their role in clearing myelin debris. Cell Rep..

[266] Masuda T., Sankowski R., Staszewski O., Bottcher C., Amann L., Sagar, Scheiwe C., Nessler S., Kunz P., van Loo G. (2019). Spatial and temporal heterogeneity of mouse and human microglia at single-cell resolution. Nature.

[267] Gudesblatt M., Bumstead B., Buhse M., Zarif M., Morrow S.A., Nicholas J.A., Hancock L.M., Wilken J., Weller J., Scott N. (2024). De-escalation of Disease-Modifying Therapy for People with Multiple Sclerosis due to Safety Considerations: Characterizing 1-Year Outcomes in 25 People Who Switched from Ocrelizumab to Diroximel Fumarate. Adv. Ther..

[268] Scolding N.J., Pasquini M., Reingold S.C., Cohen J.A., International Conference on Cell-Based Therapies for Multiple Sclerosis (2017). Cell-based therapeutic strategies for multiple sclerosis. Brain.

[269] Mullin A.P., Cui C., Wang Y., Wang J., Troy E., Caggiano A.O., Parry T.J., Colburn R.W., Pavlopoulos E. (2017). rHIgM22 enhances remyelination in the brain of the cuprizone mouse model of demyelination. Neurobiol. Dis..

[270] Nastasijevic B., Wright B.R., Smestad J., Warrington A.E., Rodriguez M., Maher J.L. (2012). Remyelination Induced by a DNA Aptamer in a Mouse Model of Multiple Sclerosis. PLoS ONE.

[271] Healy L.M., Antel J.P. (2016). Sphingosine-1-Phosphate Receptors in the Central Nervous and Immune Systems. Curr. Drug Targets.

[272] Mullershausen F., Craveiro L.M., Shin Y., Cortes-Cros M., Bassilana F., Osinde M., Wishart W.L., Guerini D., Thallmair M., Schwab M.E. (2007). Phosphorylated FTY720 promotes astrocyte migration through sphingosine-1-phosphate receptors. J. Neurochem..

[273] Osinde M., Mullershausen F., Dev K.K. (2007). Phosphorylated FTY720 stimulates ERK phosphorylation in astrocytes via S1P receptors. Neuropharmacology.

[274] Scannevin R.H., Chollate S., Jung M.Y., Shackett M., Patel H., Bista P., Zeng W., Ryan S., Yamamoto M., Lukashev M. (2012). Fumarates promote cytoprotection of central nervous system cells against oxidative stress via the nuclear factor (erythroid-derived 2)-like 2 pathway. J. Pharmacol. Exp. Ther..

[275] Noda H., Takeuchi H., Mizuno T., Suzumura A. (2013). Fingolimod phosphate promotes the neuroprotective effects of microglia. J. Neuroimmunol..

[276] Parodi B., Rossi S., Morando S., Cordano C., Bragoni A., Motta C., Usai C., Wipke B.T., Scannevin R.H., Mancardi G.L. (2015). Fumarates modulate microglia activation through a novel HCAR2 signaling pathway and rescue synaptic dysregulation in inflamed CNS. Acta Neuropathol..

[277] Ratchford J.N., Endres C.J., Hammoud D.A., Pomper M.G., Shiee N., McGready J., Pham D.L., Calabresi P.A. (2012). Decreased microglial activation in MS patients treated with glatiramer acetate. J. Neurol..

[278] Nally F.K., De Santi C., McCoy C.E. (2019). Nanomodulation of Macrophages in Multiple Sclerosis. Cells.

[279] Wang Y., Smith W., Hao D., He B., Kong L. (2019). M1 and M2 macrophage polarization and potentially therapeutic naturally occurring compounds. Int. Immunopharmacol..

[280] Kenison J.E., Jhaveri A., Li Z., Khadse N., Tjon E., Tezza S., Nowakowska D., Plasencia A., Stanton V.P., Sherr D.H. (2020). Tolerogenic nanoparticles suppress central nervous system inflammation. Proc. Natl. Acad. Sci. USA.

[281] Yeste A., Nadeau M., Burns E.J., Weiner H.L., Quintana F.J. (2012). Nanoparticle-mediated codelivery of myelin antigen and a tolerogenic small molecule suppresses experimental autoimmune encephalomyelitis. Proc. Natl. Acad. Sci. USA.

[282] Krienke C., Kolb L., Diken E., Streuber M., Kirchhoff S., Bukur T., Akilli-Ozturk O., Kranz L.M., Berger H., Petschenka J. (2021). A noninflammatory mRNA vaccine for treatment of experimental autoimmune encephalomyelitis. Science.

[283] Berek K., Bauer A., Rudzki D., Auer M., Barket R., Zinganell A., Lerch M., Hofer L., Grams A., Poskaite P. (2023). Immune profiling in multiple sclerosis: A single-center study of 65 cytokines, chemokines, and related molecules in cerebrospinal fluid and serum. Front. Immunol..

[284] Berger J.R., Markowitz C. (2018). Deciding on the Best Multiple Sclerosis Therapy: Tough Choices. JAMA Neurol..

[285] Fissolo N., Benkert P., Sastre-Garriga J., Mongay-Ochoa N., Vilaseca-Jolonch A., Llufriu S., Blanco Y., Hegen H., Berek K., Perez-Miralles F. (2024). Serum biomarker levels predict disability progression in patients with primary progressive multiple sclerosis. J. Neurol. Neurosurg. Psychiatry.

[286] Canto E., Reverter F., Morcillo-Suarez C., Matesanz F., Fernandez O., Izquierdo G., Vandenbroeck K., Rodriguez-Antiguedad A., Urcelay E., Arroyo R. (2012). Chitinase 3-like 1 plasma levels are increased in patients with progressive forms of multiple sclerosis. Mult. Scler..

[287] Perez-Miralles F., Prefasi D., Garcia-Merino A., Gascon-Gimenez F., Medrano N., Castillo-Villalba J., Cubas L., Alcala C., Gil-Perotin S., Gomez-Ballesteros R. (2020). CSF chitinase 3-like-1 association with disability of primary progressive MS. Neurol. Neuroimmunol. Neuroinflammation.

[288] Zhao H., Huang M., Jiang L. (2023). Potential Roles and Future Perspectives of Chitinase 3-like 1 in Macrophage Polarization and the Development of Diseases. Int. J. Mol. Sci..

[289] Cubas-Nunez L., Gil-Perotin S., Castillo-Villalba J., Lopez V., Solis Tarazona L., Gasque-Rubio R., Carratala-Bosca S., Alcala-Vicente C., Perez-Miralles F., Lassmann H. (2021). Potential Role of CHI3L1+ Astrocytes in Progression in MS. Neurol. Neuroimmunol. Neuroinflammation.

[290] Song Y., Jiang W., Afridi S.K., Wang T., Zhu F., Xu H., Nazir F.H., Liu C., Wang Y., Long Y. (2024). Astrocyte-derived CHI3L1 signaling impairs neurogenesis and cognition in the demyelinated hippocampus. Cell Rep..

[291] Zrzavy T., Rieder K., Wuketich V., Thalhammer R., Haslacher H., Altmann P., Kornek B., Krajnc N., Monschein T., Schmied C. (2024). Immunophenotyping in routine clinical practice for predicting treatment response and adverse events in patients with MS. Front. Neurol..

